# Optimizing sustainable healthcare location routing problem: Incorporating triage, automated medicine lockers, and soft time windows

**DOI:** 10.1371/journal.pone.0349445

**Published:** 2026-05-29

**Authors:** Zahra Samadi Bahrami, Pouria Tajasob, Seyed Mohammad Javad Mirzapour Al-e-Hashem

**Affiliations:** 1 Department of Industrial Engineering and Management Systems, Amirkabir University of Technology (Tehran Polytechnic), Tehran, Iran; 2 Institute for Transport and Logistics Management, Department of Global Business and Trade, Vienna University of Economics and Business, Vienna, Austria; University of Hong Kong, HONG KONG

## Abstract

For the past few years, pharmaceutical logistics has undergone significant changes, especially in the period referred to as the post-pandemic era, which brought major transformations to healthcare systems around the world. This research provides a novel model to enhance pharmaceutical supply chain services by routing, locating, and allocating urgent and non-urgent patients to home delivery services or automated medicine lockers. Two scenarios are proposed, with one scenario considering two types of vehicles and creating different routes to deliver medicine to automated medicine lockers or patients, and the other not distinguishing between them. The proposed mixed integer linear programming model uses a three-objective for the green open vehicle routing problem to identify the routing total costs, greenhouse gas emissions under varying speed levels due to risk of traffic congestion, and patient satisfaction. The concept of triage is also embedded into the model to prevent assigning the urgent patients to automated medicine lockers as much as possible. The problem is solved using an improved non-dominated sorting genetic algorithm-II and the LP-metric method, verified through real-world applications. Although experimental studies justify applying automated medicine lockers to cut costs significantly, 14.74% for the first scenario and 13.511% for the second, the resulted model also highlights its application to optimizing home healthcare performance. It is achieved by including greenhouse gas emissions and patient satisfaction within the framework and utilizing automated medicine lockers for pharmacy supply chain services improvement.

## Introduction

The rapid growth of home drug delivery services over the past few years has significantly improved patient care while simultaneously reducing the burden on facilities of care [1,2]. However, the COVID-19 pandemic has fundamentally transformed pharmaceutical logistics, exposing critical gaps in traditional distribution systems. Patients were forced into isolation while requiring urgent and reliable medication access, yet conventional infrastructure, dependent on in-person visits and physical facilities struggled to meet these demands efficiently [3]. This post-pandemic era has created a new reality: pharmaceutical logistics must be simultaneously responsive, sustainable, and patient-centric, requiring a fundamental rethinking of distribution network design.

The location of facilities and related transport infrastructure is a critical determining factor [4,5] that significantly influences the efficiency of emergency handling systems and pharmaceutical distribution networks. In this evolving landscape, three interconnected challenges demand urgent attention. First, economic viability remains fundamental: given elevated competitive demands, profit levels of distributors have continually fallen, making it challenging for pharmaceutical organizations to survive unless supply chains operate efficiently [6,7]. Transportation costs account for a significant portion of overall logistics expenses, and cost reduction alone is no longer sufficient for competitiveness. Second, environmental sustainability has become non-negotiable. The global commitment to combating climate change has made environmental responsibility essential [8], and logistics networks, particularly transportation operations, remain primary generators of greenhouse gas emissions [9,10]. Therefore, incorporating cost reduction along with issues of sustainability has become an essential refrain of both theoretical studies and practical applications across numerous industry spaces [11,12]. Following that, and especially critical in healthcare, rapid response and patient accessibility directly affect health outcomes. Fast delivery reduces treatment delays and enables timely intervention [13], while in-person visits to traditional facilities are often infeasible, particularly for vulnerable populations during pandemic-related quarantines or in remote areas. The COVID-19 pandemic has highlighted this need. Patients had to remain isolated, while quick and easy access to medication was essential to manage hospital capacity shortages [14].

The convergence of these three imperatives, cost efficiency, environmental sustainability, and patient responsiveness, has prompted the healthcare logistics community to explore innovative distribution models. Automated Medicine Lockers (AMLs) represent a promising solution, offering a low-contact alternative that has demonstrated significant benefits [15]. An analysis of distribution data in Helsinki showed that lockers can reduce distribution costs by 42%, while multiple studies document substantial reductions in greenhouse gas emissions [16,17]. Beyond financial and environmental gains, locker networks enhance business competitiveness and improve operational flexibility, as replenishment can be performed without customer presence and with time flexibility [18,19]. While the effectiveness of these systems depends critically on optimal location decisions, patient assignment strategies, and efficient replenishment logistics [20,21], some studies have ignored the movement of customers to the locker [22,23] or have not fully considered the effects of road network traffic [23,24].

A further dimension of this challenge involves patient stratification and triage. Not all patients have equal capacity to access self-collection lockers, differences in mobility, urgency of need, and geographic location create a heterogeneous patient population requiring differentiated service levels. Emergency patients with critical conditions may need direct home delivery despite higher costs, while stable patients can utilize locker networks. Considering the serious consequences that poor Healthcare Logistics (HCL) may have [25], this recognition necessitates a dual-service model that dynamically allocates patients based on clinical urgency while optimizing the overall network.

Recent advances in inventory management strategies provide additional support for this integrated approach. The Vendor-Managed Inventory (VMI) strategy allows sellers and buyers to coordinate fulfillment operations through regular demand reports and scheduled shipments, reducing carrying, ordering, and inventory holding costs while increasing service levels [26,27]. VMI enables the use of periodic delivery calendars aligned with patient demand patterns, supporting both locker replenishment and home delivery operations.

Despite the promise of these innovations, the performance of HCL remains highly dependent on the precision of network design and operational planning. Several critical challenges remain unaddressed: (i) the simultaneity of location-allocation decisions, sustainable routing, and fuel-efficiency optimization under real-world traffic conditions; (ii) the integration of coverage-radius-dependent service modes (AML vs. home delivery) that adapt to patient urgency; (iii) the coordination with VMI-based periodic scheduling for responsive fulfillment; and (iv) scenario analysis and evaluation of the three dimensions of sustainability in the context of a case study in realistic contexts.

This study addresses these gaps by proposing a comprehensive optimization framework for sustainable pharmaceutical distribution. The framework integrates five interdependent components: (1) Automated Medicine Locker location to establish strategic collection points; (2) patient assignment and triage based on clinical urgency and accessibility, with normal and emergency coverage radii; (3) Green Vehicle Routing Problem (GVRP) optimization for cost-effective and environmentally conscious AML replenishment; (4) home delivery services for patients outside coverage areas or requiring direct access; and (5) VMI-based periodic scheduling for coordinated, responsive fulfillment.

Recognizing that driving speed, fuel type, and traffic patterns significantly affect fuel consumption, emissions, and delivery times, our model treats these as endogenous decision variables, optimized across discrete speed levels and hourly traffic profiles. Time window constraints ensure patient satisfaction, while departure times are optimized to balance peak-hour congestion and labor costs.

The problem is formulated as a multi-objective Mixed-Integer Linear Programming (MILP) model, where solutions are compared using exact LP-metric-based approaches and an improved Non-dominated Sorting Genetic Algorithm (NSGA-II). This research addresses four key gaps: (i) simultaneity of location-allocation, green routing, and speed/fuel decisions under realistic traffic conditions; (ii) coverage-radius-dependent service modes responsive to patient clinical status; (iii) integration of VMI for periodic scheduling and operational responsiveness; and (iv) comprehensive sustainability evaluation across economic, environmental, and social dimensions through a realistic case study.

The paper is structured as follows: Section 2 provides a critical literature review; Section 3 presents the MILP formulation; Section 4 describes the improved NSGA-II and LP-metric framework; Section 5 reports case study results; Section 6 presents sensitivity analyses and managerial insights; and Section 7 provides conclusions and future directions.

## Literature review

Location-Routing Problems (LRPs) are a combination of two VRPs and Facility Location Problems (FLPs) [28]. By simultaneously considering both facility location and routing planning, higher quality and more efficient results can be achieved [29]. Veenstra et al. [30] introduced an LRP in healthcare facilities in the Netherlands. In this case, medicine delivery from a local pharmacy can be done through lockers, where patients are within the locker’s coverage radius, or patients can receive their medicines through the home delivery option. Suwatcharachaitiwong et al. [31] continued the study of Veenstra et al. [29] and investigated a multiple delivery problem in HCL, which aims to find the best location for installing lockers. While Veenstra et al. [30] and Suwatcharachaitiwong et al. [31] introduced locker-based medicine distribution in healthcare, their models lack environmental objectives, triage-based patient allocation, and variable-speed routing, all critical for post-pandemic pharmaceutical logistics. Moreover, there are some recent LRP studies in HCL [6,32,33]. The LRP problem utilizes mathematical models and optimization to minimize the traveled distance, total travel time, number of vehicles, and earliness and tardiness penalties, while also maximizing the transportation cost function and customer satisfaction.

The term GVRP was first used by Erdoğan and Miller-Hooks [34] for the Green VRP routing of alternative fuel vehicles like electric vehicles. Zhao et al. [35] proposed a two-stage method that combines a simulated annealing algorithm and an attention mechanism approach to solve a Green LRP (GLRP) with shared pickup stations, optimizing electric vehicle routes and station locations to minimize costs under capacity constraints. While Liao et al. [36] formulated a multi-objective GVRP considering customer satisfaction for the food distribution industry, their model does not account for healthcare-specific features such as patient triage, differentiated coverage radii based on patient urgency, or AML-based medication distribution. Furthermore, their study does not consider open-loop routing, fuel-type-dependent emissions, or the integration of departure time as a decision variable. Our model extends this line of research by incorporating all of these features simultaneously, thereby providing a more comprehensive and realistic framework tailored to pharmaceutical logistics. Xu et al. [37] presented a non-linear multi-objective GVRP model to optimize customer satisfaction and fuel consumption based on time-varying speed.

The model under our study aims to reduce GHG emissions and fuel consumption, which, in addition to the distance traveled, takes into account factors such as the type of fuel consumed, vehicle weight, and road slope at different speeds. Masmoudi et al. [38] investigated a transport routing model with heterogeneous transport fleets in home health services. Their model had a trade-off between drivers’ wages and GHG emissions. The model with a heterogeneous fleet outperformed the homogeneous fleet when the results were also compared. Van Montfort et al. [39] integrated task-splitting into home healthcare routing and scheduling by proposing optimization models and heuristics to reduce operational costs and improve resource allocation. However, these studies [38,39] primarily focus on general home healthcare logistics and do not consider pharmaceutical-specific constraints such as patient triage, differentiated coverage radii, or the integration of AMLs for distribution. Asghari and Mirzapour Al-e-Hashem [40] addressed a green Pick-up and Delivery Problem (PDP) for home hemodialysis machines. Dukkanci et al. [41] proposed a GLRP by adjusting the speed in each arc so that customers are served in the corresponding time windows. Mirzapour Al-e-hashem et al. [42] modeled an ambulance routing problem in the organ transplant supply chain, considering traffic congestion. Zhang et al. [43] considered an optimization model for green vehicle scheduling and routing problems with different variable speeds, with additional wages in non-working periods and soft time window constraints. The results show that the departure time affects the car’s fuel consumption and CO2 emission, and the optimal departure time saves fuel consumption and reduces CO2 emission. Therefore, the departure time was assumed as a variable to calculate its optimal value. In addition, the drivers’ wages were calculated based on normal and abnormal working hours. Adelhütte et al. [44] addressed the minimization of delays in non-emergency patient transport by modeling it as a dynamic vehicle routing problem with general time windows, solved via an MILP approach adaptable to real-time updates and additional constraints. Vosooghi et al. [45] presented a scenario-based redesign of a relief supply-chain network by considering humanitarian constraints and triage.

Another point to be considered in transportation problems is whether there is an obligation to return the vehicle to the origin or whether the vehicle is free of return obligation (OVRP). Due to the development of third-party companies, the outsourcing of distribution operations, and the concept of the sharing economy, OVRP problems have become more prominent in recent literature. Niu et al. [46] modeled an open GVRP. They compared the results with the closed model, and in the OVRP, the costs were 20% lower.

Locating facilities or lockers and allocating customers are usually associated with the coverage radius. Zhen et al. [47] investigated an OVRP model in the postal industry. Their model considered the coverage radius for attracting and dispatching vehicles. Their purpose was to determine the appropriate radius. Jabali et al. [10] examined a VRP model to serve customers allocated to distribution facilities with a determined coverage radius. Enthoven et al. [48] considered a two-echelon routing model for the postal industry as a hybrid linear mathematical model. Sitek et al. [49] presented a capacitated routing model considering PDP and time windows. The problem was to find the optimal points of the postal lockers and the best route for the distribution of packages. Zang et al. [15] introduced a parcel locker routing problem with a compensation-based reward strategy, formulated it as an MILP and set-partitioning models, and solved it using a Branch-and-Price (B&P) algorithm with a customized labeling approach to optimize last-mile delivery. Dumez et al. [50] and Mancini and Gansterer [51] studied a VRP model with delivery options as well as lockers for last-mile delivery during the corresponding time window. Yu et al. [52] extended the electric VRP with time windows with partial recharges by incorporating parcel lockers as a self-pickup option, proposed a mathematical model and an Adaptive Large Neighborhood Search (ALNS) algorithm to minimize delivery costs, and evaluated performance through benchmark instances and managerial analysis. Grabenschweiger et al. [53] analyzed heterogeneous locker boxes in their studies. Liu et al. [54] demonstrated that a hybrid Q-learning-network-based method can outperform exact methods and GA for the mobile parcel locker problem. However, their work addresses a single-objective optimization for mobile locker routing, whereas our framework requires simultaneous multi-objective optimization of cost, GHG emissions, and patient satisfaction. Multi-objective RL-based methods are less mature than single-objective approaches and typically require complex reward shaping; consequently, NSGA-II remains the most established and practical choice for multi-objective location-routing problems in the literature. While RL-based enhancements could be explored in future research, NSGA-II is well-suited to this problem.

While parcel lockers have been extensively studied in e-commerce last-mile delivery [48,50,51,53], their application in healthcare, specifically as AMLs remains largely unexplored. Veenstra et al. [30] and Suwatcharachaitiwong et al. [31] are among the few studies that considered locker-based medicine distribution, but neither incorporated environmental objectives, patient triage, or variable-speed routing. The concept of AMLs extends beyond conventional parcel lockers by requiring compliance with pharmaceutical storage standards, patient-specific allocation based on clinical urgency, and integration with healthcare scheduling systems. The limited attention to AMLs in the literature represents a significant gap, given the demonstrated cost savings in general locker-based distribution [16] and the growing demand for contactless healthcare delivery solutions post-pandemic.

Another aspect of the related literature is to pay attention to the multiplicity or singularity of considering facilities such as vehicles or distribution centers, decision variables, periods, goods, or objective functions. Ghasemi et al. [55] have proposed a multi-period multi-vehicle MIP model for the earthquake response phase, which includes location, routing, and allocation (ALRP). Algendi et al. [56] proposed a multi-objective MILP model for home healthcare staffing, routing, and scheduling over a multi-day horizon, addressing real-world constraints and demonstrating improved solution efficiency through model enhancements. Reihaneh and Ghoniem [57] investigated routing with a demand allocation problem that optimizes on-time delivery locations and routes vehicle sites that use a central depot. Guo and Bard [58] developed a three-step algorithm for scheduling home healthcare providers, balancing travel, workload, and continuity constraints, and demonstrated through real-world data that it significantly improves nurse and patient schedules compared to existing practices. Zhao et al. [59] introduced a bi-objective MILP model for home health care routing and scheduling under service time uncertainty, solved using a stochastic ALNS embedded in an enhanced multi-directional local search framework, with results demonstrating its efficiency and robustness through real-life application and sensitivity analysis. Although these studies address various multi-objective home healthcare scheduling and routing problems [56,59], none integrate AML-based distribution with triage-responsive routing and fuel-dependent emissions simultaneously, which are key aspects of our current study. Baradaran et al. [60] studied a multi-objective model considering customer satisfaction, travel time, and transportation costs with heterogeneous fleets and solved it with the bee colony algorithm. Akbari et al. [61] developed an Integer Programming (IP) model and a metaheuristic algorithm to solve the home healthcare routing and scheduling problem with prioritized patients, aiming to minimize total weighted waiting time. The methods were evaluated on benchmark instances and a real-world case study involving COVID-19 patients in Istanbul. Tricoire et al. [62] presented a bi-objective covering tour model with the costs of opening distribution centers and the routing cost for vehicle fleets. In their model, depending on the distance, 1% of the customers go from their homes to the nearest distribution centers. Li et al. [63] proposed a MILP model and an improved NSGA-II algorithm to solve a home healthcare scheduling problem with coordinated caregiver and vehicle access. Results show the approach reduces costs and supports efficient planning.

Although the delivery of medications has been increasingly implemented, recent literature reviews indicate that only a few studies have addressed the logistics problem of medication delivery by simultaneously determining the locations of potential facilities and vehicle routing.

In summary, the existing literature exhibits the following gaps: (1) no study has jointly addressed AML location, patient allocation based on triage, and green open vehicle routing in a pharmaceutical context; (2) speed and fuel-type decisions remain disconnected from patient satisfaction and GHG emission objectives in most models; (3) the open-loop routing structure, which reflects real-world outsourcing practices, has not been combined with locker-based distribution in healthcare; and (4) departure time optimization accounting for traffic-dependent wage structures is absent from prior pharmaceutical LRP models. The present study addresses all four gaps simultaneously.

Our work distinguishes itself by proposing a comprehensive Allocating-Locating Green Open Vehicle Routing Problem (ALGOVRP) that integrates AML location optimization, triage-responsive patient allocation, and green routing with variable speeds and fuel-type considerations. Unlike prior pharmaceutical logistics studies, our multi-objective framework simultaneously minimizes cost and GHG emissions while maximizing patient satisfaction, incorporates departure time as a decision variable to optimize fuel consumption, and calculates driver wages based on traffic-dependent hours. By combining AML-based distribution with home delivery options and considering differentiated coverage radii for emergency versus routine patients, we provide a more holistic and realistic framework for post-pandemic pharmaceutical logistics.

Table 1 presents a comparative analysis of our proposed ALGOVRP model against existing literature on location-routing, green vehicle routing, and healthcare logistics. This table highlights the unique contribution of our work in simultaneously addressing AML location decisions, triage-based patient allocation, open-loop routing, multi-objective optimization (cost, GHG emissions, and patient satisfaction), fuel-type and speed-dependent routing with variable departure times, and driver wage structures, attributes that no prior study has collectively incorporated. The notation (*) in Table 1 indicates that fuel consumption and GHG emissions are calculated according to the formula presented in Turkensteen [64], demonstrating our adherence to established emission calculation methods while extending them to the pharmaceutical logistics context.

**Table 1:** A summary of the selected recent papers.

Reference	Health Care	Triage	Locker	Time Window	Fleet Type	Speed	Flow	Case	Fuel Cons. & Green Factors				Modeling & Resolution Particularities				
									S	D	*	E	Objective(s)	Problem Type	Modeling	Exact	Heuristic & Metaheuristic
[30]	✓	–	✓	–	Het.	–	CL	HCL	–	–	–	–	ECO	ALRP	MLP	B&B	Hyb.
[6]	✓	–	–	Hard	Hom.	–	CL	HCL	–	–	–	–	SOC	LRP	MILP	LR/ B&C	–
[41]	–	–	–	Hard	Hom.	Var.	CL	Gnl.	–	–	✓	–	ECO	LRP	MIP	–	ILS
[65]	–	–	–	Hard	Het.	Fix	HOL	Gnl.	✓	✓	✓	–	ENV	RP	DP	B&P	–
[48]	–	–	–	–	Het.	–	CL	LMD	–	–	–	–	ECO	ARP	MIP	–	ALNS
[31]	✓	–	✓	–	Het.	–	CL	HCL	–	–	–	–	ECO	ALRP	MLP	–	GA
[66]	–	–	–	–	Hom.	Fix	OL	Gnl.	–	–	–	–	ECO	ALRP	MILP	–	VNS/ICA
[53]	–	–	✓	Hard	Hom.	–	CL	LMD	–	–	–	–	ECO	RP	MLP	–	ALNS
[51]	–	–	✓	Hard	Hom.	–	CL	LMD	–	–	–	–	ECO	RP	MLP	–	LNS
[67]	✓	–	–	Hard	Hom.	Var.	CL	EMS	–	–	–	–	SOC	ALRP	INLP	B&B	–
[68]	–	–	–	–	Het.	–	OL	PPSC	–	–	–	–	- ECO - ENV - SOC	ALRP	NLP	–	MGWO
[63]	✓	–	–	Soft	Hom.	Fix	CL	HHC	–	–	–	–	- ECO - SOC	RP	MILP	–	NSGA-II
[61]	✓	✓	–	–	Hom.	Fix	CL	HCL	–	–	–	–	- SOC	RP	IP	–	VNS
[59]	✓	–	–	Soft	Het.	–	CL	HCL	–	–	–	–	- ECO - SOC	RP	MILP	–	ALNS
[56]	✓	✓	–	Hard	Het.	–	CL	HCL	–	–	–	–	- ECO - SOC	RP	MILP	–	Heuristic
[44]	✓	–	–	Soft	Hom.	–	CL	HCL	–	–	–	–	- SOC	RP	MILP	–	Heuristics
Present study	✓	✓	✓	Hyb.	Het. & Hom.	Var.	OL	HCL	✓	✓	✓	✓	- ECO - ENV - SOC	ALRP	MILP	LP	NSGA-II

**ALRP:** Allocating-Location-Routing Problem; **ALNS:** Adaptive Large Neighborhood Search; **B&B:** Branch and Bound; **B&C:** Branch and Cut; **B&P:** Branch and Price; **CL:** Closed Loop; **Cons.:** Consumption; **D:** Distance; **DP:** Dynamic Programming; **E:** Emission; **ECO:** Economic Objective; **ENV:** Environmental Objective; **EMS:** Emergency Medical Service; **GA:** Genetic Algorithm; **Gnl.:** General; **HCL:** Health Care Logistics; **Het.:** Heterogeneous; **HHC:** Home Health Care; **HOL:** Half Open Loop; **Hom.:** Homogeneous; **Hyb.:** Hybrid; **ICA:** Independent Component Analysis; **ILS:** Iterated Local Search; **INLP:** Integer NonLinear Programming **IP:** Integer Programming; **LMD:** Last-Mile Delivery; **LP:** LP-Metric; **LR:** Lagrangian Relaxation; **LRP:** Location-Routing Problem; **MD:** Meal Delivery; **MGWO:** Modified Grey Wolf Optimization; **MILP:** Mixed Integer Linear Programming; **MINLP:** Mixed Integer NonLinear Programming; **MIP:** Mixed Integer Programming; **NLP:** NonLinear Programming; **OL:** Open Loop; **PPSC:** Perishable Products Supply Chain; **RP:** Routing Proble; **S:** Speed; **SOC:** Social Objective; **TF:** Type of Fuel; **Var.:** Variable; **VNS:** Variable Neighborhood Search.

## Problem definition and formulation

**Fig 1:**
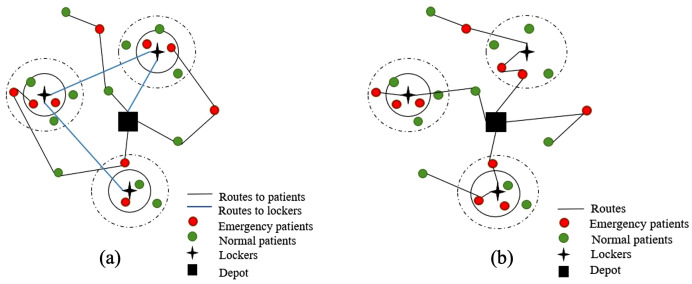
Samples of the first scenario and the second scenario.

For the problem addressed in this study, a central warehouse is considered for medicine distribution. AMLs can be visited at pre-determined and installed potential points. Therefore, one of the goals is to find the optimal locations for choosing AMLs for replenishment. In this study, the aim is to identify the optimal combination of AML selection for stocking and allocating patients within the coverage radius of the AMLs, as well as to determine the most efficient route for medicine delivery to the AMLs and for home delivery to patients outside the coverage radius. It is assumed that these two routes are traveled by two types of vehicles, separately from the central warehouse, and under two scenarios. In the first scenario, the paths for refilling the AMLs and transferring medicine to the patients are separate. This separation of routes has happened for several reasons: the drivers who visit the patients should have better social and pharmaceutical information. Another reason is dealing with patients and increasing the service time. Therefore, the cost of drivers will be different. In the second scenario, there is one type of vehicle, and it is possible to visit every patient and every selected AML consecutively. Two types of patients, namely normal and emergency patients, were considered under the concept of triage. This means that, based on historical data related to each patient and the type of required medicine, it is assumed that some patients have a higher priority for receiving their medicine on time, as well as a different type of service. Since some emergency patients are unable to walk to the selected AML and collect their medicine, they can only be served through the home delivery option. Accordingly, the coverage radius of AMLs is categorized into normal radius (for patients under normal conditions) and emergency radius (for patients under emergency conditions). If an AML is selected for replenishment and a patient is within the radius, this patient will be allocated to it; otherwise, the patient will be served through home delivery. A penalty cost is assumed for the system for each emergency patient assigned to an AML, encouraging the system to prioritize home medicine delivery for emergency patients whenever possible. Considering the soft time window in this problem helps to respond to the patients at a specific time based on their condition. As shown in Fig 1a, two types of routes can be considered: one route from the depot to the selected AMLs and another route from the depot to the patients who are not within the coverage radius. Note that there may be some emergency patients who are within the emergency radius (circle drawn with a full line), but they will be served directly by home delivery because of their penalty cost (the worse the condition of the patient and the more urgent the situation, the higher the penalty). Fig 1b shows the second scenario in which there is no difference among routes, meaning that all vehicles can visit patients or AMLs on the same route.

An integral part of each distance traveled is the amount of fuel consumed by the vehicle, according to the type of fuel formulated in the first objective function. The cost of drivers’ wages has also been included according to normal and abnormal working hours. In addition, the cost of tolls has been proposed, so considering the second objective function simultaneously, the shortest route is not necessarily the least expensive route. In the second objective function, GHG emissions produced by vehicles are included, based on the amount and type of fuel consumed as well as vehicle speed, with the goal of minimizing these emissions. Departure time is also considered a decision variable, and by optimizing or removing fixed departure time requirements, the system can reduce drivers’ wage costs incurred during traffic hours. A binary variable is assumed to determine the optimal speed that selects the appropriate speed level for each arc. By considering the speed variable, this capability is added to the model to calculate the reach time to the nodes according to the selected speed. Since the problem is planned for patients in HCL, it is critical to determine the emergency radius of the AMLs so that the allocation of an emergency patient to the AML is done if the location is only a short distance away or the determined penalty cost is minor due to the patient’s condition. The customer satisfaction level function is formulated regarding medicine delivery time and combined soft time windows in the third objective function. Therefore, the third objective function helps us to optimize the patients’ satisfaction levels. In addition, the model is formulated as an open-loop system, allowing vehicles to be free from the restriction of returning to the depot after completing their routes. Since distributing companies may use rental vehicles or third-party companies, formulating the model as OVRP is more realistically implementable.

### Notation

**Sets**

O: Depot.
O+1: The destination node.
$P^{n}$: Normal patients.
$P^{e}$: Emergency patients.
*L*: AMLs.
K: Vehicles related to the delivery operation to patients (home delivery).
M: vehicles related to the delivery operation to AMLs.
$K^{\prime }$: Total fleet vehicles ${(K}^{\prime }=K\cup M)$.
R: Defined speed levels.
tf: Fuel types.

**Parameters**

$a^{\prime }$: Start of working time.
$b^{\prime }$: End of working time.
$\overset{n}{\underset{j}{r}}$: Normal coverage radius of AML j for the normal patients.
$s_{i}$: Service time for the patient i.
$\overset{\prime }{\underset{j}{s}}$: Service time for the AML j.
${ELT}_{i}$: The upper bound of the time that the patient i will be able to receive the medicine.
${EET}_{i}$: The lower bound of the time that patient i will be able to receive the medicine.
${PLT}_{i}$: The upper bound of the time that the patient i prefers to receive the medicine.
${PET}_{i}$: The lower bound of the time that patient i prefers to receive the medicine.
$d_{ij}$: The distance between node i and node j.
$c_{w}$: Driver’s wage during normal hours.
$c_{a}$: Driver’s wage during irregular hours.
${{\bar{v}}_{ij}}^{k^{\prime }}$: The average speed of the vehicle $k^{\prime }$ during the movement of the arc (i,j).
${fc}^{tf}$: The unit cost of fuel type tf.
$\overset{k^{\prime }r}{\underset{ij}{F}}$: Fuel consumption function for the vehicle $k^{\prime }$ with the level speed of r during arc (i,j).
${ef}^{{CO}_{\text{2}},tf}$: Emission coefficient, the amount of CO_2_ emitted per unit of fuel consumed of type tf*.*
${tl}_{ij}$: The cost of road tolls along the arc (i,j)*.*
$\overset{k^{\prime }}{\underset{ij}{Uv}}$: The upper bound of vehicle speed $k^{\prime }$ along an arc (i,j).
$v^{r}$: The value of the speed level r∈R.
$t_{w}$: The lower bound of routine time to work.
$t_{a}$: The upper bound of routine time to work.
${pc}_{i}$: Penalty cost of allocating the emergency patient i to an AML.
${ic}_{j}$: The cost of installing AML j*.*

**Variables**

$w_{i}$: A binary variable that determines whether the patient i will be served by a vehicle directly.
$z_{i}$: A binary variable that determines whether the patient i is assigned to the AML.
$u_{i,j}$: A binary variable that determines whether the patient i is allocated to AML j.
$v_{i}$: A binary variable that determines whether AML i is installed or not.
$x_{ijk}$: A binary variable that is equal to 1 if the vehicle k travels from patient i to patient j.
$\overset{\prime }{\underset{ijm}{x}}$: A binary variable that is equal to 1 if the vehicle m travels from AML i to AML j.
$y_{ik}$: A binary variable that shows if the node i is assigned to the vehicle k to visit.
$\overset{\prime }{\underset{i,m}{y}}$: A binary variable that shows if the node i is assigned to the vehicle m to visit.
$\overset{\prime k^{\prime },r}{\underset{ij}{w}}$: A binary variable that determines the vehicle $k^{\prime }$ travels arc (i,j) with a speed level of r.
$\delta^{tf}$: A binary variable that determines if the fuel type of tf is selected, is equal to 1.
$t_{ik}$: The arrival time of the vehicle k at the node i.
$\overset{\prime }{\underset{jm}{t}}$: The arrival time of the vehicle m at the node j*.*
$\overset{k^{\prime }}{\underset{o}{\lambda }}$: Start time of operation for the vehicle $k^{\prime }$.
$\overset{k^{\prime }}{\underset{n+\text{1}}{\lambda }}$: Finish time of operation for the vehicle $k^{\prime }$.
$\overset{e}{\underset{j}{r}}$: The size of the emergency coverage radius for AML j.

### Formulation

The first objective function aims to minimize costs and considers the economic dimension. So, the cost of fuel consumption, tolls, and drivers’ wages has been calculated, which is as equation (1).


$$\begin{array}{cc} MinZ\text{1}= & \sum_{k^{\prime }\in K^{\prime }}\sum_{i\in O\cup P^{n}\cup P^{e}\cup L}\sum_{j\in O+\text{1}\cup P^{n}\cup P^{e}\cup L}{fc}^{tf}.\delta^{tf,k^{\prime }}.d_{ij}.\overset{k^{\prime }r}{\underset{ij}{F}}.\overset{k^{\prime }}{\underset{ij}{x}}\\ & +\sum_{k^{\prime }\in K^{\prime }}G(c_{w},c_{a},\overset{k^{\prime }}{\underset{n+\text{1}}{\lambda }},\overset{k^{\prime }}{\underset{o}{\lambda }})+\sum_{k^{\prime }\in K^{\prime }}\sum_{i\in O\cup P^{n}\cup P^{e}\cup L}\sum_{j\in O+\text{1}\cup P^{n}\cup P^{e}\cup L}{tl}_{ij}.\overset{k^{\prime }}{\underset{ij}{x}}\\ & +\sum_{j\in L}{ic}_{j}.v_{j}+\sum_{i\in P^{e},j\in L}{pc}_{j}.u_{ij} \end{array}$$ (1)

**Fig 2:**
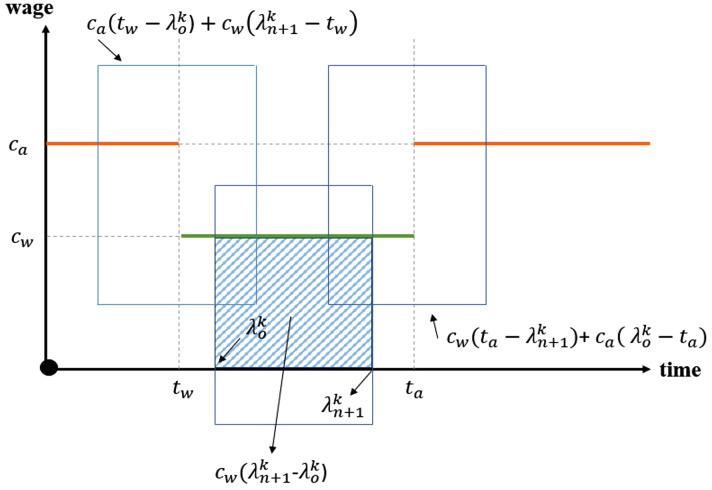
The visualization of function G.

The first term represents the cost of fuel consumption of the type tf during $d_{ij}$ distance at the unit cost of ${fc}^{tf}$, if the vehicle $k^{\prime }\in K^{\prime }$ travels the arc (i,j). The second term is related to the total cost of drivers’ wages. In this function, two types of working hours are considered: normal hours, which are the routine working periods for drivers, and irregular hours, which occur due to traffic or special time intervals during which it is more difficult to operate. The function G is presented in constraint (23) and is shown in Fig 2, so wages are calculated according to the hours they work. Calculating the cost of tolls for the routes is the third term. The fourth term represents the installation cost of AMLs. The fifth term is pertinent to the penalty cost of servicing emergency patients by allocating them to an AML and not by home delivery. The second objective function is to reduce the GHG emissions. Our model incorporates vehicle weight and road slope directly into the calculation of fuel consumption and, consequently, GHG emissions for each segment of a vehicle’s route. This is achieved through the adoption of the Comprehensive Modal Emission Model (CMEM) [69,70], first adapted to a VRP by Bektaş and Laporte [71] aimed to minimize travel distance by recognizing that the greenest route isn’t always the shortest.

So that if the vehicle $k^{\prime }\in K^{\prime }$ uses fuel type tf and the emission effect of fuel type tf is ${ef}^{{CO}_{\text{2}},tf}$ per unit of the traveled distance, the total GHGs emitted in the distance $d_{ij}$ and with the fuel consumption $\overset{k^{\prime }r}{\underset{ij}{F}}$ will be formulated in equation (2).


$$\begin{array}{c} MinZ\text{2}=\sum_{i\in O\cup P^{n}\cup P^{e}\cup L}\sum_{j\in O+\text{1}\cup P^{n}\cup P^{e}\cup L}\sum_{k^{\prime }\in K^{\prime }}\sum_{tf\in tf}\delta^{tf,k^{\prime }}.{ef}^{{CO}_{\text{2}}}.d_{ij}.\overset{k^{\prime }r}{\underset{ij}{F}}.\overset{k^{\prime }}{\underset{ij}{x}} \end{array}$$ (2)

The calculation of the required fuel consumption, which is a function of the vehicle speed, and to avoid the high complexity of the problem, historical data was used in the study, and the speed on each arc was assumed to be a constant average value, as presented in Table 2.

**Table 2:** Model parameters.

Notation	Description	Typical values
Re	Engine speed (rev/s)	36.67
Ve	Engine displacement (liters)	6.9
Lf	Engine friction factor (kJ/rev/L)	0.20
A	Frontal surface area (m^2^)	8.0
$c_{d}$	Coefficient of aerodynamic drag	0.7
$c_{r}$	Coefficient of rolling resistance	0.01
ξ	Fuel-to-air mass ratio	1
τ	Acceleration (m/s^2^)	0
$n_{tf}$	Vehicle drive train efficiency	0.45
η	Efficiency parameter for diesel engines	0.45
Ke	Heating value of a typical diesel fuel (kJ/g)	44
ψ	Conversion factor (g/s to L/s)	737
g	Gravitational constant (m/s^2^)	9.81
ρ	Air density (kg/m^3^)	1.2041


$$\begin{array}{c} F\left(\nu_{r}\right)=\lambda \left(kf.N.\upsilon +\omega \lambda \alpha .\nu_{r}+\beta \gamma .\overset{\text{3}}{\underset{r}{\nu }}\right).d/\nu_{r} \end{array}$$ (3)

Where $\lambda =\frac{\xi }{Ke.\psi }$, $\gamma =\frac{\text{1}}{\text{1}\text{0}\text{0}\text{0}.n_{tf}.\eta }$,$\alpha =\tau +g.c_{r}$, and $\beta =\text{0}.\text{5}.c_{d}.\rho .A$. For arc (i,j), the amount of fuel consumption for vehicle $k^{\prime }\in K^{\prime }$ at speed r will be as equation (4).


$$\begin{array}{c} \overset{k^{\prime }r}{\underset{ij}{F}}=\overset{k^{\prime },r}{\underset{i,j}{w^{\prime }}}\lambda \left(kf.N.\upsilon +\omega \lambda \alpha .\nu_{r}+\beta \gamma .\overset{\text{3}}{\underset{r}{\nu }}\right).d_{ij}/\nu_{r} \end{array}$$ (4)

According to CMEM, fuel consumption depends factors in equation 4, where $\alpha =\tau +g.c_{r}$ (includes road slope via rolling resistance) and the vehicle weight is embedded in the curb weight parameter. This means that: (1) heavier vehicles consume more fuel per unit distance due to increased rolling resistance; (2) road slope affects the gravitational component of fuel consumption, uphill routes consume significantly more fuel than flat routes of the same distance; and (3) speed affects fuel consumption non-linearly (aerodynamic drag scales with v3). Therefore, two routes of identical distance can have vastly different emission profiles depending on vehicle load, terrain, and speed.

**Fig 3:**
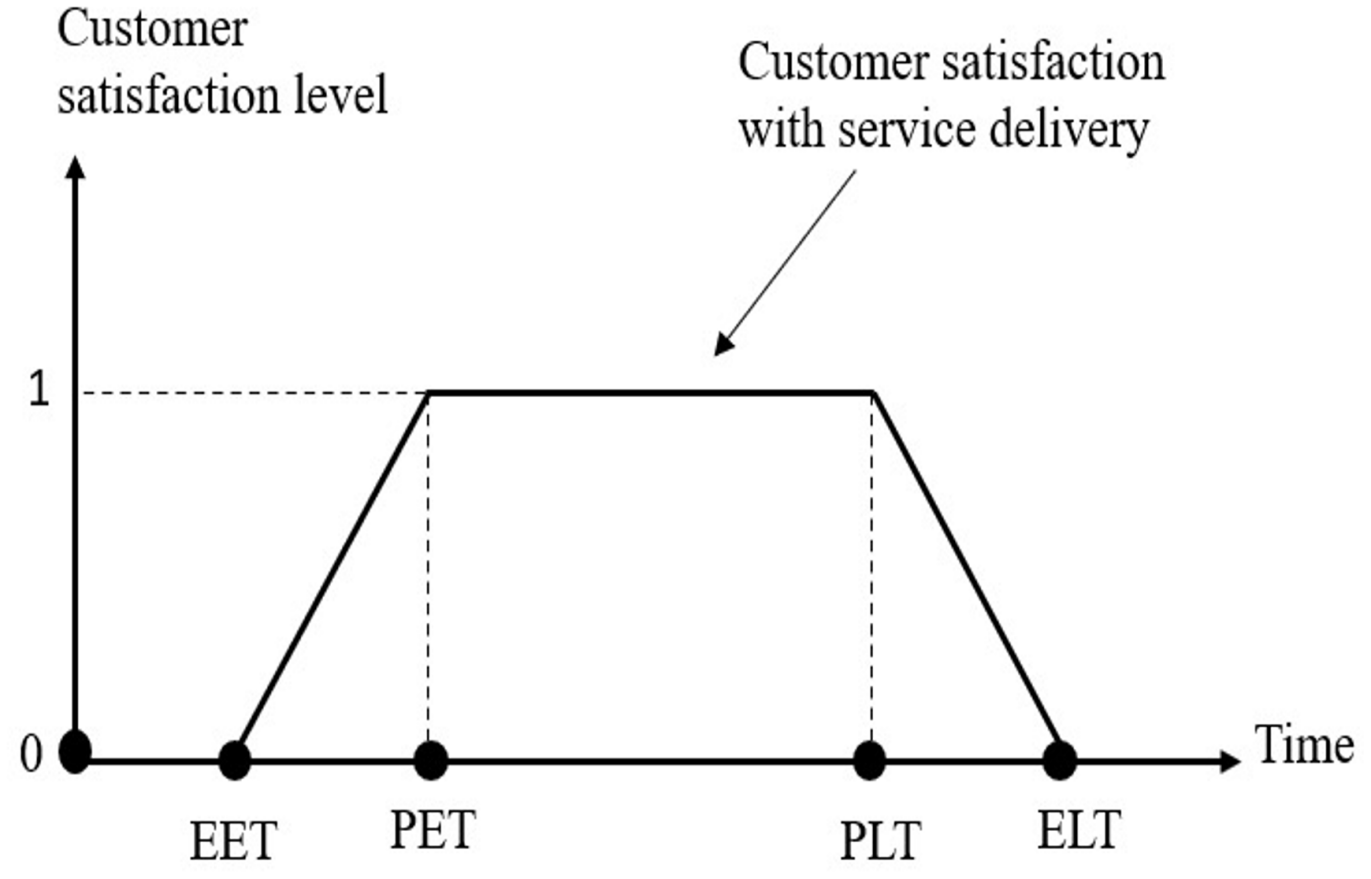
The relationship between customer satisfaction and preferable (𝐏𝐄𝐓,𝐏𝐋𝐓) and endurable (𝐄𝐄𝐓,𝐄𝐋𝐓) time windows for patients.


In the third objective function, the model seeks to increase the satisfaction level of patients according to the delivery time. Regarding to the study of Xu et al. [37], and As shown in Fig 3, two types of soft time windows are defined for each patient. The preferable time window (PET,PLT) represents the interval in which the patient ideally wishes to receive the medicine; if the vehicle arrives within this window, the satisfaction level equals 1. The endurable time window (EET,ELT) represents the maximum tolerable range beyond which patient satisfaction drops to zero. Specifically, if the vehicle arrives before EET or after ELT, the satisfaction is zero. Between EET and PET, satisfaction increases linearly from 0 to 1. Between PLT and ELT, satisfaction decreases linearly from 1 to 0. Finally, for the third objective function, the model includes the following:

$$\begin{array}{c} {Cs}^{p}\left(t_{ik}\right)=\left\{ \begin{array}{c} @l\text{0}\;t_{ik}<EETort_{ik}>ELT\\ \frac{t_{ik}-EET}{PET-EET}\;EET<t_{ik}<PET\\ \text{1}\;PET\leq t_{ik}<PLT\\ \frac{ELT-t_{ik}}{ELT-PLT},\;PLT\leq t_{ik}\leq ELT \end{array}\right. \end{array}$$ (5)

It should be noted that for the patients allocated to AMLs, since there is no time limitation for the patient’s presence to receive the medicine, and patients can visit the AML whenever they want, only the upper bound of time windows is applied. This means that if the AML is filled up to the upper bound of the preferred time of the patient (PLT), the patient’s utility will be equal to one, and if the AML is filled between PLT and ELT, the utility value will decrease linearly until it reaches zero. If this value exceeds the upper bound of the endurable time window (ELT) for receiving the medicine, the utility will set to zero.


$$\begin{array}{c} {Cs}^{l}\left({t^{\prime }}_{jm}\right)=\left\{ \begin{array}{c} @l\text{1}\;{t^{\prime }}_{jm}\leq PLT\\ \frac{ELT-{t^{\prime }}_{jm}}{ELT-PLT}\;PLT\leq {t^{\prime }}_{jm}\leq ELT\\ \text{0}\;{t^{\prime }}_{jm}>ELT \end{array}\right. \end{array}$$ (6)

Therefore, the total amount of patient satisfaction, according to the previously defined functions, will be the summation of the satisfaction level of those who are within a coverage radius of AMLs or are served through the depot directly.


$$\begin{array}{c} MaxZ\text{3}=\sum_{i\in P^{n}\cup P^{e}}\sum_{k\in K}{Cs}^{p}(t_{ik})+\sum_{i\in P^{n}\cup P^{e}}\sum_{k\in K}{Cs}^{l}(\overset{\prime }{\underset{jm}{t}}) \end{array}$$ (7)

The final problem modeling is as follows:

$\begin{array}{c} \begin{array}{c} MinZ\text{1}=\sum_{k^{\prime }\in K^{\prime }}\sum_{i\in O\cup P^{n}\cup P^{e}\cup L}\sum_{j\in O+\text{1}\cup P^{n}\cup P^{e}\cup L}{fc}^{tf}.\delta^{tf,k^{\prime }}.d_{ij}.\overset{k^{\prime }r}{\underset{ij}{F}}.\overset{k^{\prime }}{\underset{ij}{x}}\\ \;\;\;\;+\sum_{k^{\prime }\in K^{\prime }}G(c_{w},c_{a},\overset{k^{\prime }}{\underset{n+\text{1}}{\lambda }},\overset{k^{\prime }}{\underset{o}{\lambda }})+\sum_{k^{\prime }\in K^{\prime }}\sum_{i\in O\cup P^{n}\cup P^{e}\cup L}\sum_{j\in O+\text{1}\cup P^{n}\cup P^{e}\cup L}{tl}_{ij}.\overset{k^{\prime }}{\underset{ij}{x}}\\ \;\;\;\;+\sum_{j\in L}{ic}_{j}.v_{j}+\sum_{i\in P^{e},j\in L}{pc}_{j}.u_{ij} \end{array} \end{array}$


$$\begin{array}{c} MinZ\text{2}=\sum_{i\in O\cup P^{n}\cup P^{e}\cup L}\sum_{j\in O+\text{1}\cup P^{n}\cup P^{e}\cup L}\sum_{k^{\prime }\in K^{\prime }}\sum_{tf\in tf}\delta^{tf,k^{\prime }}.{ef}^{{CO}_{\text{2}}}.d_{ij}.\overset{k^{\prime }r}{\underset{ij}{F}}.\overset{k^{\prime }}{\underset{ij}{x}} \end{array}$$

$$\begin{array}{c} MaxZ\text{3}=\sum_{i\in P^{n}\cup P^{e}}\sum_{k\in K}{Cs}^{p}(t_{ik})+\sum_{i\in P^{n}\cup P^{e}}\sum_{k\in K}{Cs}^{l}(\overset{\prime }{\underset{jm}{t}}) \end{array}$$

St:


$$\begin{array}{c} \sum_{j\in L}u_{ij}=z_{i}\;\forall i\in P^{n}\cup P^{e} \end{array}$$ (8)

$$\begin{array}{c} w_{i}+z_{i}=\text{1}\;\forall i\in P^{n}\cup P^{e} \end{array}$$ (9)

$$\begin{array}{c} u_{ij}\leq v_{j}\;\forall i\in P^{n}\cup P^{e},j\in L \end{array}$$ (10)

$$\begin{array}{c} u_{ij}.d_{ij}\leq \overset{n}{\underset{j}{r}}\;\forall i\in P^{n},j\in L \end{array}$$ (11)

$$\begin{array}{c} u_{ij}.d_{ij}\leq \overset{e}{\underset{j}{r}}\;\forall i\in P^{e},j\in L \end{array}$$ (12)

$$\begin{array}{c} \overset{e}{\underset{j}{r}}.v_{j}\leq d_{ij}+z_{i}M\;\forall i\in P^{e},j\in L \end{array}$$ (13)

$$\begin{array}{c} \overset{n}{\underset{j}{r}}.v_{j}\leq d_{ij}+z_{i}M\;\forall i\in P^{n},j\in L \end{array}$$ (14)

$$\begin{array}{c} \sum_{i\in P^{n}\cup P^{e}\cup O}x_{ijk}=\sum_{i\in P^{n}\cup P^{e}\cup O}x_{jik}=\overset{k}{\underset{i}{y}}\;\forall j\in P^{n}\cup P^{e},k\in K \end{array}$$ (15)

$$\begin{array}{c} \sum_{i\in L\cup O}\overset{\prime }{\underset{ijm}{x}}=\sum_{i\in L\cup O}\overset{\prime }{\underset{jim}{x}}=\overset{m}{\underset{i}{y^{\prime }}}\;\forall j\in L,m\in M \end{array}$$ (16)

$$\begin{array}{c} x_{oik}=x_{jo^{\prime }k}=\overset{k}{\underset{o}{y}}\;\forall i\in P^{n}\cup P^{e},k\in K \end{array}$$ (17)

$$\begin{array}{c} \overset{\prime }{\underset{ojm}{x}}=\overset{\prime }{\underset{jo^{\prime }m}{x}}=\overset{m}{\underset{o}{y^{\prime }}}\;\forall j\in L,m\in M \end{array}$$ (18)

$$\begin{array}{c} \sum_{k}\overset{k}{\underset{i}{y}}=w_{i}\;\forall i\in P^{n}\cup P^{e} \end{array}$$ (19)

$$\begin{array}{c} \sum_{m}\overset{m}{\underset{j}{y^{\prime }}}=\nu_{j}\;\forall j\in L \end{array}$$ (20)

$$\begin{array}{c} x_{ii^{\prime }k}(t_{ik}+s_{i}+\sum_{r\in R}\left(\frac{d_{ii^{\prime }}}{\nu^{r}}\right)\overset{kr}{\underset{ii^{\prime }}{w}}-t_{i^{\prime }k}\leq \text{0}\forall i\in P^{n}\cup P^{e}\cup O,i^{\prime }\in P^{n}\cup P^{e},k\in K \end{array}$$ (21)

$$\begin{array}{c} \overset{\prime }{\underset{jj^{\prime }m}{x}}(\overset{\prime }{\underset{jm}{t}}+\overset{\prime }{\underset{j}{s}}+\sum_{r\in R}\left(\frac{d_{jj^{\prime }}}{\nu^{r}}\right)\overset{mr}{\underset{jj^{\prime }}{w}}-\overset{\prime }{\underset{j^{\prime }m}{t}}\leq \text{0}\forall j\in L\cup O,j^{\prime }\in L,m\in M \end{array}$$ (22)

$$\begin{array}{c} G\left(c_{w},c_{a},\overset{k^{\prime }}{\underset{n+\text{1}}{\lambda }},\overset{k^{\prime }}{\underset{o}{\lambda }}\right)=\left\{ \begin{array}{c} @l\sum_{k^{\prime }}c_{w}(\overset{k^{\prime }}{\underset{n+\text{1}}{\lambda }}-t_{w})+c_{a}\left(t_{w}-\overset{k^{\prime }}{\underset{o}{\lambda }}\right),\;\overset{k^{\prime }}{\underset{o}{\lambda }}\leq t_{w}\leq \overset{k^{\prime }}{\underset{n+\text{1}}{\lambda }}\leq t_{a}\\ \sum_{k^{\prime }}c_{a}(\overset{k^{\prime }}{\underset{n+\text{1}}{\lambda }}-\overset{k^{\prime }}{\underset{o}{\lambda }}-(t_{a}-t_{w}))+c_{w}(t_{a}-t_{w}),\;\overset{k^{\prime }}{\underset{o}{\lambda }}\leq t_{w}\leq t_{a}\overset{k^{\prime }}{\underset{n+\text{1}}{\leq \lambda }}\\ \sum_{k^{\prime }}c_{w}(\overset{k^{\prime }}{\underset{n+\text{1}}{\lambda }}-\overset{k^{\prime }}{\underset{o}{\lambda }}),),\;t_{w}\leq \overset{k^{\prime }}{\underset{o}{\lambda }}\leq \overset{k^{\prime }}{\underset{n+\text{1}}{\lambda }}\leq t_{a}\\ \sum_{k^{\prime }}c_{w}(t_{a}-\overset{k^{\prime }}{\underset{o}{\lambda }})+c_{a}\left(\overset{k^{\prime }}{\underset{n+\text{1}}{\lambda }}-t_{a}\right),),\;t_{w}\leq \overset{k^{\prime }}{\underset{o}{\lambda }}\leq t_{a}\leq \overset{k^{\prime }}{\underset{n+\text{1}}{\lambda }} \end{array}\right. \end{array}$$ (23)

$$\begin{array}{c} \overset{k^{\prime }}{\underset{o}{\lambda }}\geq a^{\prime }\;\forall k^{\prime }\in K^{\prime } \end{array}$$ (24)

$$\begin{array}{c} \overset{k^{\prime }}{\underset{n+\text{1}}{\lambda }}\leq b^{\prime }\forall k^{\prime }\in K^{\prime } \end{array}$$ (25)

$$\begin{array}{c} \sum_{r}\overset{rk^{\prime }}{\underset{ij}{w}}=\text{1}\;\forall k^{\prime }\in K^{\prime },(i,j)\in A \end{array}$$ (26)

$$\begin{array}{c} \sum_{r}\nu_{r}.\overset{rk^{\prime }}{\underset{ij}{w}}\leq U\overset{k^{\prime }}{\underset{ij}{\nu }}\;\forall k^{\prime }\in K^{\prime },(i,j)\in A \end{array}$$ (27)

$$\begin{array}{c} \overset{n}{\underset{j}{r}}\geq \overset{e}{\underset{j}{r}}\;\forall j\in L \end{array}$$ (28)

$$\begin{array}{c} \overset{m}{\underset{i}{y^{\prime }}},\overset{rk^{\prime }}{\underset{ij}{w}},\overset{k}{\underset{i}{y}},x_{ijk},\overset{\prime }{\underset{ijk}{x}},\nu_{i},u_{ij},z_{i},w_{i}\in \left\{\text{0},\text{1}\right\}\overset{k^{\prime }}{\underset{n+\text{1}}{\lambda }},\overset{k^{\prime }}{\underset{o}{\lambda }},t_{ik},\overset{\prime }{\underset{im}{t}}\geq \text{0} \end{array}$$ (29)

**Location- and allocation-related constraints.** Constraints (8)-(14) and (28) define the facility-location and allocation decisions. Constraint (8) states that if patient i is allocated to an AML, it should only be allocated to one AML. Constraint (9) states that each patient i can be served in only one way (by AML or home delivery). Constraint (10) states that patient i is allocated to AML j only if AML j is opened. Constraint (11) ensures that a normal patient i is allocated to AML j only if the distance $d_{ij}$ between them does not exceed the normal coverage radius $\overset{n}{\underset{j}{r}}$. Constraint (12) ensures that an emergency patient i is allocated to AML j only if the distance $d_{ij}$ between them does not exceed the emergency coverage radius $\overset{e}{\underset{j}{r}}$. Constraints (13)-(14) are the complementary big-M linearization of the coverage–allocation relationship. Constraint (13) states that if an emergency patient i is served by home delivery ($z_{i}=\text{1}$), the distance constraint is relaxed; otherwise, the emergency radius of the activated AML j must not exceed the distance $d_{ij}$, preventing infeasible allocations when the AML opening variable $v_{j}$ is active. Constraint (14) applies the same logic for normal patients. Constraint (28) indicates that the normal radius should be greater than the emergency radius.

**Routing- and flow balance constraints.** Constraints (15)-(22) govern vehicle routing operations. Constraint (15) expresses the flow balance to determine the route from patient i to patient j by vehicle k. It should be noted that both emergency and normal patients can be accessed by vehicle k. Constraint (16), as in the previous case, determines the path created to deliver the medicine to AMLs by vehicle m. Constraint (17) expresses the flow balance constraint by vehicle k for the warehouse. Constraint (18) also considers the flow balance for the warehouse and is intended to connect the warehouse and the set of AML nodes. Constraint (19) states that only nodes of patients i are visited by the vehicle k that are assigned to the warehouse, and constraint (20) states that only nodes of AMLs that are decided to be filled are visited. Constraint (21) is related to determining the time window. If the decision is made on the movement of vehicle k on arc (i,j), the sequence of these two nodes should be expressed through the traveled times for arc (i,j), and the service time to point i. It is considered that this sequence will also contribute to sub-tour elimination. In the same way, constraint (22) expresses the time window for visiting each of the AMLs by vehicle m.

**Wage, Time, and Speed Constraints.** Constraints (23)-(27) govern driver wage calculation, working time bounds, and speed level assignment. Constraint (23) expresses the driver’s wage calculation. The earlier the drivers start working and the later they finish their tour, the costs will increase due to the irregular time wages. Constraints (24) and (25) express the hard time window for the depot, which is the daily working time, and all of the activities should be started and finished within this period. Constraints (26) and (27) are related to determining the speed level to travel from point i to j for each vehicle. Also, each vehicle should travel arc (i,j) with only a determined speed level, which concerns constraint (26), and constraint (27) determines that speed should not exceed a certain level, which is variable according to the selected route.

**Variable domains**. Constraint (29) is related to binary and continuous variables.

The mathematical formulation for the second scenario is available in S1 File – Section A.

### Linearization

#### Linearization of the multiplication of a continuous variable by a binary variable.

To summarize, a general linearization formula is presented for each part of the model that has been formulated in this manner. For the linearization of the multiplication of continuous variables by binary variables, the following formulation is provided, illustrated through constraints (21) and (22) related to time windows as an example.


$$\begin{array}{c} \mu_{ijk}=x_{ijk}.t_{ik} \end{array}$$ (30)

$$\begin{array}{c} \mu_{ijk}\leq t_{ik}\;\forall i\in P^{n}\cup P^{e}\cup O,j\in P^{n}\cup P^{e},k\in K \end{array}$$ (31)

$$\begin{array}{c} \mu_{ijk}\leq M.x_{ijk}\;\forall i\in P^{n}\cup P^{e}\cup O,j\in P^{n}\cup P^{e},k\in K \end{array}$$ (32)

$$\begin{array}{c} \mu_{ijk}\geq t_{ik}-M.\left(\text{1}-x_{ijk}\right)\;\forall i\in P^{n}\cup P^{e}\cup O,j\in P^{n}\cup P^{e},k\in K \end{array}$$ (33)

$$\begin{array}{c} \mu_{ijk}\geq \text{0} \end{array}$$ (34)

The same steps are applied for multiplying the routing variable by the speed level variable. Additionally, the constraint related to the emergency radius is a product of the binary variable $\upsilon_{\text{1}}$ in the continuous variable related to the emergency radius and will be linearized the same way. In addition, for the first part of the first objective function, which is to minimize the fuel consumption cost, and in the second objective function, to minimize the GHG emissions, in which $\overset{k^{\prime }}{\underset{ij}{x}}$ is multiplied by $\overset{rk^{\prime }}{\underset{ij}{w}}$formulated in the fuel consumption function $\overset{k^{\prime }r}{\underset{ij}{F}}$, the same approach will be applied to linearize, as well. Section B of the S1 File presents the details of the linearization for these parts and other ones that need to be linearized.

#### Linearization of piecewise functions.

In the described mathematical model, the two functions related to the calculation of drivers’ wages and patients’ satisfaction are among the piecewise functions that must be linearized. The linearization method is explained generally, and the drivers’ wages calculation function is used as an example; the patient satisfaction function will be linearized using the same method. The G function calculates the driver’s wage cost in the first objective function. Therefore, this function is equal to one of the criteria based on the working hours. This equality has been shown as two smaller and larger constraints. This concept is expressed by the pairs of constraints (35) and (36), (37) and (38), (39) and (40), (41) and (42). The rest of the constraints state the conditions of each criterion. For instance, the first criterion of function G is stated with constraints (43), (44), and (45). Constraints (44) and (45) state that the first criterion must be followed if the time of finishing work is less than $t_{a}$, or at the normal hour, and constraint (43) states that this is the case if the driver’s departure time from the depot is less than $t_{w}$ (overtime). This is accomplished by including a binary variable in constraint (55). Only one binary variable should be selected among others according to constraint (54).


$$\begin{array}{c} G(c_{w},c_{a},\overset{k^{\prime }}{\underset{n+\text{1}}{\lambda }},\overset{k^{\prime }}{\underset{o}{\lambda }})\geq \sum_{k^{\prime }}c_{w}(\overset{k^{\prime }}{\underset{n+\text{1}}{\lambda }}-t_{w})+c_{a}(t_{w}-\overset{k^{\prime }}{\underset{o}{\lambda }})-M(\text{1}-\upsilon_{\text{1}}) \end{array}$$ (35)

$$\begin{array}{c} G(c_{w},c_{a},\overset{k^{\prime }}{\underset{n+\text{1}}{\lambda }},\overset{k^{\prime }}{\underset{o}{\lambda }})\leq \sum_{k^{\prime }}c_{w}(\overset{k^{\prime }}{\underset{n+\text{1}}{\lambda }}-t_{w})+c_{a}(t_{w}-\overset{k^{\prime }}{\underset{o}{\lambda }})+M(\text{1}-\upsilon_{\text{1}}) \end{array}$$ (36)

$$\begin{array}{c} G(c_{w},c_{a},\overset{k^{\prime }}{\underset{n+\text{1}}{\lambda }},\overset{k^{\prime }}{\underset{o}{\lambda }})\geq \sum_{k^{\prime }}c_{w}(\overset{k^{\prime }}{\underset{n+\text{1}}{\lambda }}-\overset{k^{\prime }}{\underset{o}{\lambda }})-M(\text{1}-\upsilon_{\text{2}}) \end{array}$$ (37)

$$\begin{array}{c} G(c_{w},c_{a},\overset{k^{\prime }}{\underset{n+\text{1}}{\lambda }},\overset{k^{\prime }}{\underset{o}{\lambda }})\leq \sum_{k^{\prime }}c_{w}(\overset{k^{\prime }}{\underset{n+\text{1}}{\lambda }}-\overset{k^{\prime }}{\underset{o}{\lambda }})+M(\text{1}-\upsilon_{\text{2}}) \end{array}$$ (38)

$$\begin{array}{c} G(c_{w},c_{a},\overset{k^{\prime }}{\underset{n+\text{1}}{\lambda }},\overset{k^{\prime }}{\underset{o}{\lambda }})\geq \sum_{k^{\prime }}c_{w}(t_{a}-\overset{k^{\prime }}{\underset{o}{\lambda }})+c_{a}(\overset{k^{\prime }}{\underset{n+\text{1}}{\lambda }}-t_{a})-M(\text{1}-\upsilon_{\text{3}}) \end{array}$$ (39)

$$\begin{array}{c} G(c_{w},c_{a},\overset{k^{\prime }}{\underset{n+\text{1}}{\lambda }},\overset{k^{\prime }}{\underset{o}{\lambda }})\leq \sum_{k^{\prime }}c_{w}(t_{a}-\overset{k^{\prime }}{\underset{o}{\lambda }})+c_{a}(\overset{k^{\prime }}{\underset{n+\text{1}}{\lambda }}-t_{a})+M(\text{1}-\upsilon_{\text{3}}) \end{array}$$ (40)

$$\begin{array}{c} G\left(c_{w},c_{a},\overset{k^{\prime }}{\underset{n+\text{1}}{\lambda }},\overset{k^{\prime }}{\underset{o}{\lambda }}\right)\geq \sum_{k^{\prime }}c_{a}(\overset{k^{\prime }}{\underset{n+\text{1}}{\lambda }}-\overset{k^{\prime }}{\underset{o}{\lambda }}-(t_{a}-t_{w}))+c_{w}(t_{a}-t_{w})-M(\text{1}-\upsilon_{\text{4}}) \end{array}$$ (41)

$$\begin{array}{c} G(c_{w},c_{a},\overset{k^{\prime }}{\underset{n+\text{1}}{\lambda }},\overset{k^{\prime }}{\underset{o}{\lambda }})\leq \sum_{k^{\prime }}c_{a}(\overset{k^{\prime }}{\underset{n+\text{1}}{\lambda }}-\overset{k^{\prime }}{\underset{o}{\lambda }}-(t_{a}-t_{w}))+c_{w}(t_{a}-t_{w})+M(\text{1}-\upsilon_{\text{4}}) \end{array}$$ (42)

$$\begin{array}{c} \overset{k^{\prime }}{\underset{o}{\lambda }}\leq t_{w}+M(\text{1}-\upsilon_{\text{1}}) \end{array}$$ (43)

$$\begin{array}{c} \overset{k^{\prime }}{\underset{n+\text{1}}{\lambda }}\geq t_{w}-M(\text{1}-\upsilon_{\text{1}}) \end{array}$$ (44)

$$\begin{array}{c} \overset{k^{\prime }}{\underset{n+\text{1}}{\lambda }}\leq t_{a}+M(\text{1}-\upsilon_{\text{1}}) \end{array}$$ (45)

$$\begin{array}{c} \overset{k^{\prime }}{\underset{o}{\lambda }}\geq t_{w}-M(\text{1}-\upsilon_{\text{2}}) \end{array}$$ (46)

$$\begin{array}{c} \overset{k^{\prime }}{\underset{n+\text{1}}{\lambda }}\leq \overset{k^{\prime }}{\underset{n+\text{1}}{\lambda }}+M(\text{1}-\upsilon_{\text{2}}) \end{array}$$ (47)

$$\begin{array}{c} \overset{k^{\prime }}{\underset{n+\text{1}}{\lambda }}\leq t_{a}+M(\text{1}-\upsilon_{\text{2}}) \end{array}$$ (48)

$$\begin{array}{c} \overset{k^{\prime }}{\underset{o}{\lambda }}\geq t_{w}-M(\text{1}-\upsilon_{\text{3}}) \end{array}$$ (49)

$$\begin{array}{c} \overset{k^{\prime }}{\underset{o}{\lambda }}\leq t_{a}+M(\text{1}-\upsilon_{\text{3}}) \end{array}$$ (50)

$$\begin{array}{c} \overset{k^{\prime }}{\underset{n+\text{1}}{\lambda }}\geq t_{a}-M(\text{1}-\upsilon_{\text{3}}) \end{array}$$ (51)

$$\begin{array}{c} \overset{k^{\prime }}{\underset{o}{\lambda }}\leq t_{w}+M(\text{1}-\upsilon_{\text{4}}) \end{array}$$ (52)

$$\begin{array}{c} \overset{k^{\prime }}{\underset{n+\text{1}}{\lambda }}\geq t_{a}-M(\text{1}-\upsilon_{\text{4}}) \end{array}$$ (53)

$$\begin{array}{c} \upsilon_{\text{1}}+\upsilon_{\text{3}}+\upsilon_{\text{3}}+\upsilon_{\text{4}}=\text{1} \end{array}$$ (54)

$$\begin{array}{c} \upsilon_{i}\in \left\{\text{0},\text{1}\right\} \end{array}$$ (55)

## Solution methodology

### LP-metric method

The LP-metric method is one of the Multi-Criteria Decision-Making (MCDM) methods that solve the Multi-Objective Decision-Making (MODM) models [72]. By combining multiple objective functions into a single one, as done in the study by Mirzapour Al-e-Hashem et al. [73], the method minimizes the total (e.g., squared) relative deviations of each objective from its optimal value. For this purpose, the sum of the relative deviations of the objective functions from their optimal values should be minimized. Consequently, the objective function is defined as follows:


$$\begin{array}{c} MinZ=\sum_{i=\text{1}}^{n}w_{i}{\left(\frac{\overset{*}{\underset{i}{z}}-z_{i}}{\overset{*}{\underset{i}{z}}}\right)}^{p} \end{array}$$ (56)

$$\begin{array}{c} g_{i}\left(x_{i}=x_{\text{1}},x_{\text{2}},\ldots ,x_{n}\right)\leq bj=\text{1},\text{2},\ldots ,m \end{array}$$ (57)

$$\begin{array}{c} z_{i}=f_{i}(x_{\text{1}},x_{\text{2}},\ldots ,x_{n}) \end{array}$$ (58)

$$\begin{array}{c} x_{i}\geq \text{0},\;z_{i}=free \end{array}$$ (59)

Expression (56) is a comprehensive measure of the importance (weight) of the $i^{th}$ goal. To eliminate the problem of the different scales of the objectives, the deviation of the optimal solution of the $i^{th}$ objective is divided by $\overset{*}{\underset{i}{z}}$. P specifies the degree of emphasis on deviations in such a way that the larger this value is, the greater the emphasis will be on the biggest deviation. However, it should be admitted that the development of exact methods on LRP is unfortunately slow. Most of the articles that consider an exact method only deploy it to verify the proposed mathematical model using a commercial solver, such as CPLEX [74].

### Improved NSGA-II

**Fig 4:**
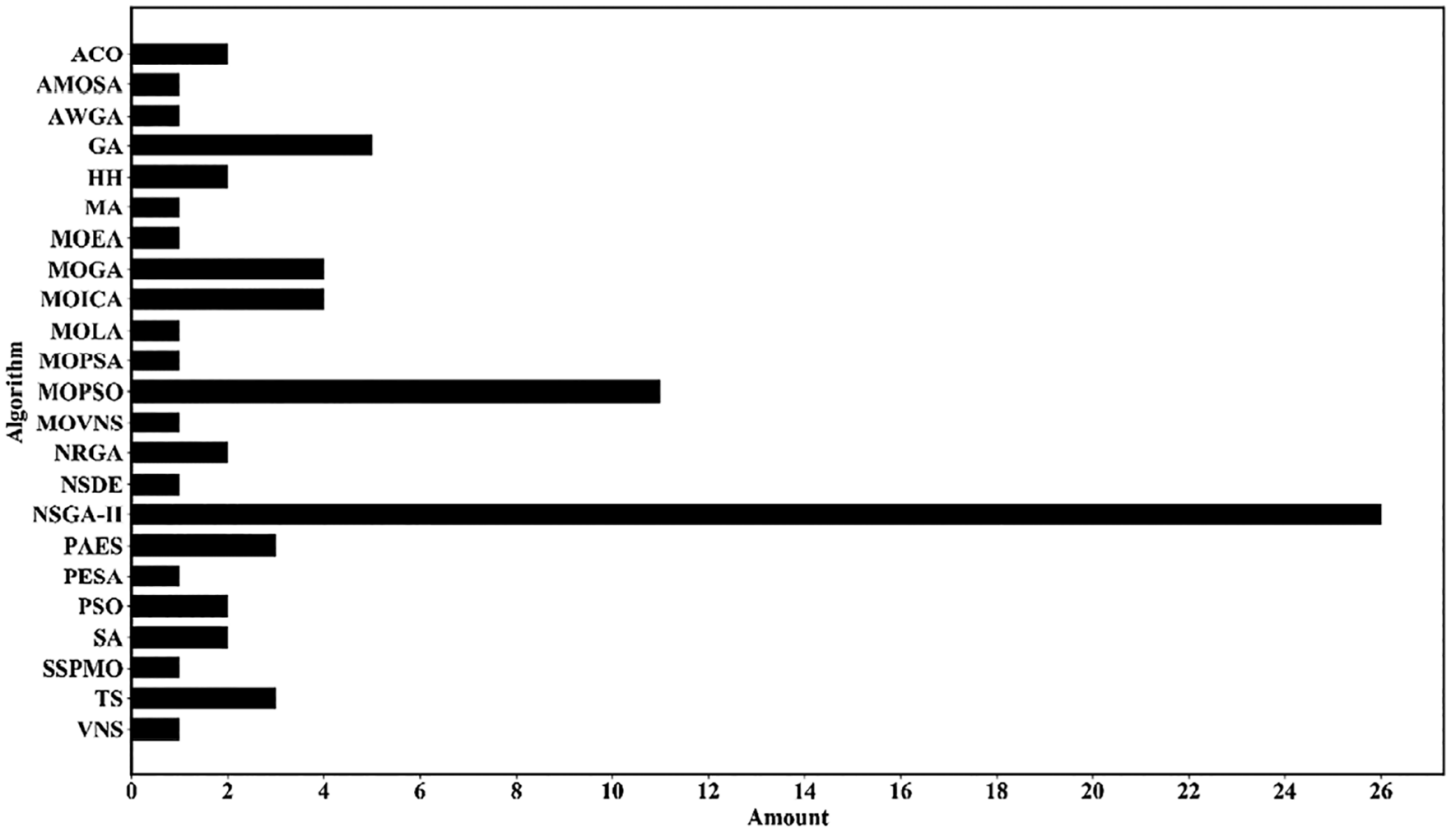
Metaheuristic algorithms for multi-objective problems.

To date, metaheuristics are still the most popular option to solve an LRP model [74] due to their effectiveness and efficiency [75]. According to the investigations of Mara et al. [74], NSGA-II is the preferred framework for multi-objective locating routing problems. As shown in Fig 4, the NSGA-II algorithm has the most usage among 222 academic papers published.

**Fig 5:**
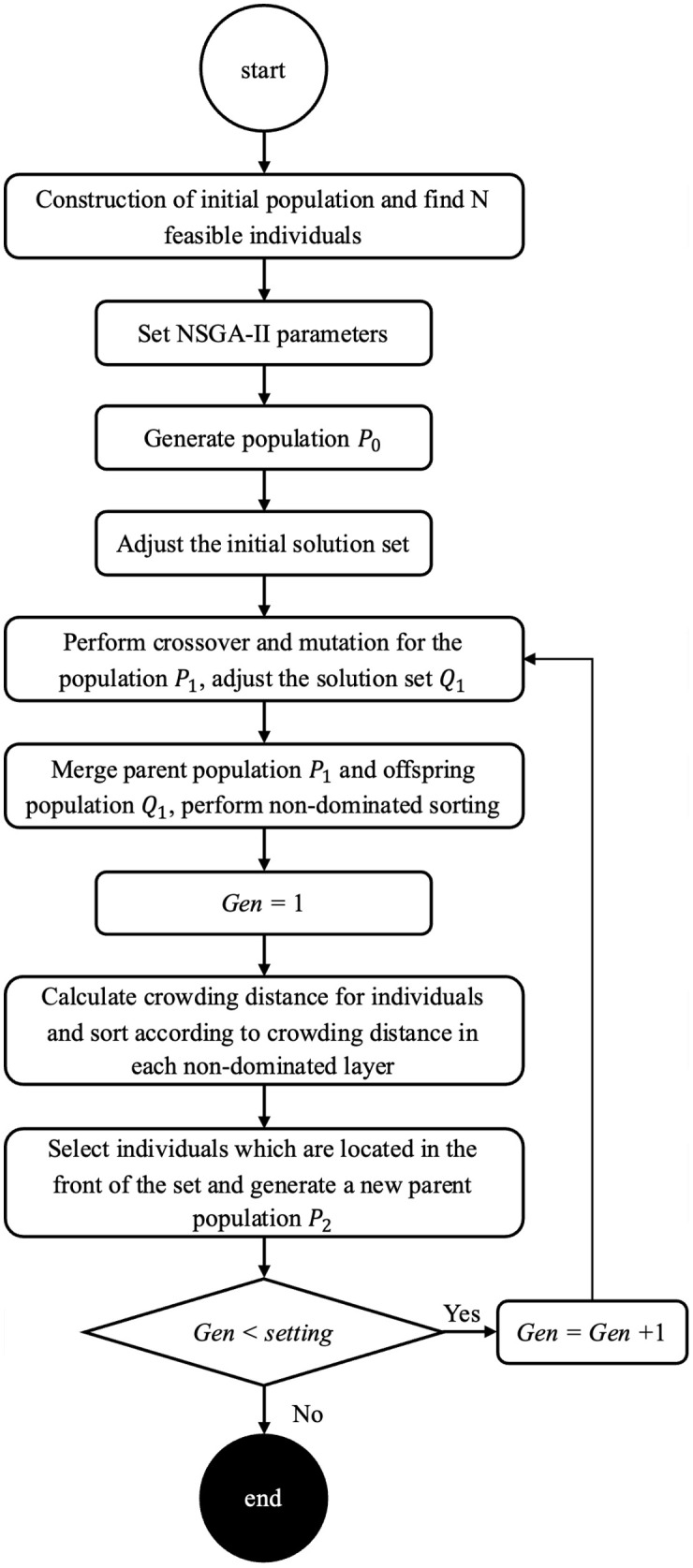
Flow of the customized NSGA-II.

In this study NSGA-II is customized to find the best solutions. The steps of proposed NSGA-II are shown in Fig 5 to solve the problem. In the following sections, the details of each component of the algorithm are thoroughly explained.

#### Chromosome representation.

**Fig 6:**
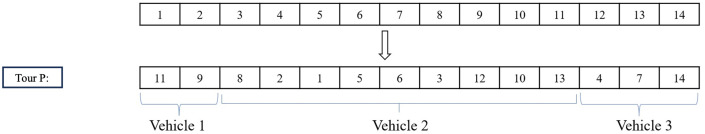
Chromosome representation with 3 vehicles and 14 customers.

In this problem, all selected AMLs form a set named Tour L, and all patients not assigned to an AML form a set as Tour P, and are defined by a chromosome consisting of a set of sections, which represent the route of a vehicle. According to the number of vehicles, each Tour P/Tour L is randomly divided among the vehicles. In one solution, as shown in Fig 6, Tour P there are 14 patients and 3 vehicles to visit them. And each path is displayed as a string: k1=[11,9], k2=[8,2,1,5,6,3,12,10,13], k3=[4,7,14].

**Fig 7:**
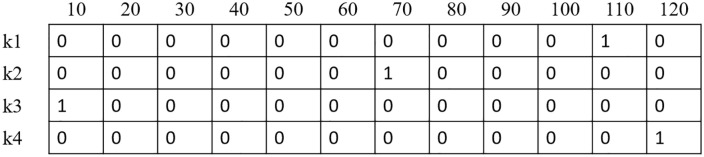
Chromosome representation with 4 vehicles and 12 speed levels.

Moreover, the chromosome of speed is in the form of multidimensional matrices. In such a way that there is only one 1 in each row and the other values are equal to 0. Each row of the matrix corresponds to a vehicle and the columns represent the speed levels and the next two dimensions are related to the route. For instance, a chromosome of speed for 12 levels from 10 to 120 (km/h) for 4 vehicles for arc (i,j) is shown in Fig 7. The chromosomes related to other variables are the same.

#### Population initialization with greedy strategies.

**Fig 8:**
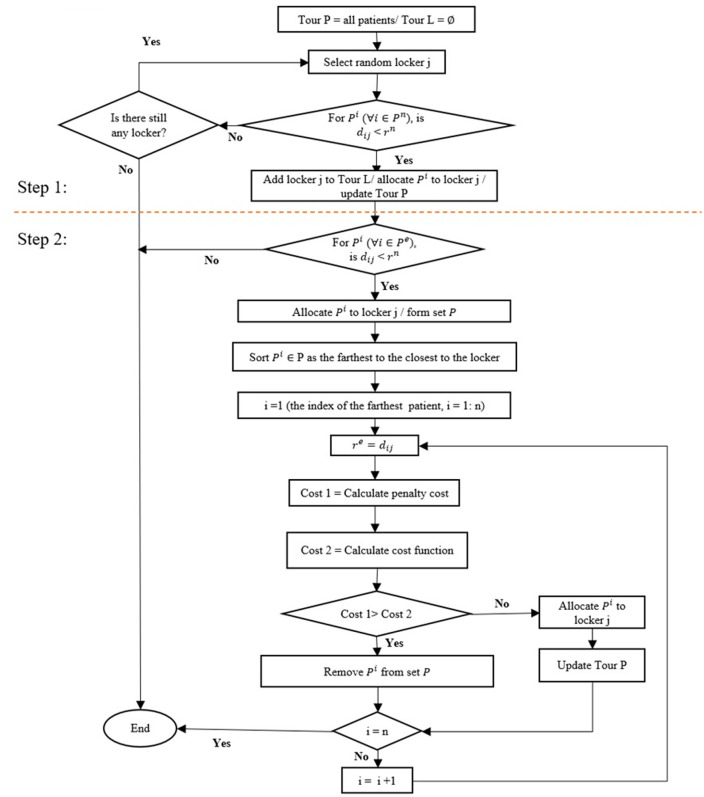
Population initialization.


In Fig 8, the steps of initializing the population through the greedy strategy for the allocation of emergency patients are illustrated. A greedy strategy is used to initialize the population in two steps. In the first step, normal patients are allocated to AMLs. And in the next step, it will be decided whether emergency patients will be served via AMLs or home delivery. At first, it is assumed that Tour L=∅, and all patients are included in Tour P. Then, by choosing AMLs randomly and allocating normal patients to them, AMLs will be added to Tour L, and patients will be removed from Tour P. In the second step, it is assumed that all emergency patients within the normal radius are assigned to the AML and form the set P. These patients are then sorted based on their distance from the AML, with the farthest patient being selected for analysis first. So, the emergency radius equals the distance of the farthest patient to the AML. Then, by removing the farthest patient from the set P, and choosing it for home delivery service, the algorithm calculates whether the cost function will be reduced rather to the penalty cost or not. So, if the cost function is reduced by not assigning the patient to the AML, the patient is removed from the set P. This process continues until all patients are selected. Note that higher penalty costs are assigned to patients with higher priority levels. So, for these patients, medicine delivery will be done by home delivery. In order to calculate the cost function, the first objective in equation (1) is modified as expression (60):

$$\begin{array}{c} \text{Cost function}=\sum_{k^{\prime }}\sum_{i}\sum_{j}{fc}^{tf}.\delta^{tf,k^{\prime }}.d_{ij}.\overset{k^{\prime }r}{\underset{ij}{F}}.\overset{k^{\prime }r}{\underset{ij}{x}}+\sum_{k^{\prime }}G(c_{w},c_{a},\overset{k^{\prime }}{\underset{n+\text{1}}{\lambda }},\overset{k^{\prime }}{\underset{o}{\lambda }})+\sum_{k^{\prime }}\sum_{i}\sum_{j}{tl}_{ij}.\overset{k^{\prime }}{\underset{ij}{x}} \end{array}$$ (60)

#### Crossover and mutation operators.

**Fig 9:**
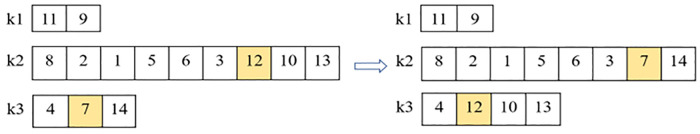
Crossover operator.

**Fig 10:**
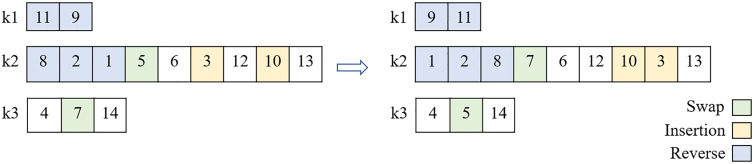
Mutation operators.

The single-point method was used for the crossover operation. As shown in Fig 9, in this way, a random number between 2 and n−1 (n is the length of each route) is chosen as the intersection point (yellow units) and the strings are replaced. However, since this may damage the gene (particularly in vehicles assigned to two patients), a gene repair function is employed. This function aims to avoid losing a solution. For this purpose, only the last unit of a route is allowed to be an intersection point. Furthermore, three mutation methods, swap, inversion, and insertion are randomly used as operators. Fig 10 illustrates these operators.

#### NS algorithm and parent selection strategy.

Two important factors should be considered in solving multi-objective optimization problems: quality and order. A Pareto front is sought that simultaneously ensures high solution quality and maximum compatibility with the fitness function. The members of the Pareto front are regularly and uniformly located. The NSGA-II algorithm proposes non-dominant sorting and crowding distance for this purpose. The NS algorithm involves two important parameters:

• $S_{P}$: The set of members of the population that are defeated by p.
• $n_{p}$: The number of times p is defeated by others.

And the steps of the NS algorithm are described as follows:

1. For all population members, put: $S_{P}=\{\}$ and $n_{p}=\text{0}$.
2. For each member of the population such as p, and each member of the population such as q:
3. If p dominates q, add q to $S_{P}$.
4. If q dominates p, add one to $n_{p}$.
5. Add all members of the population where $n_{p}=\text{0}$ to $F_{\text{1}}$.
6. Set the counter of the fronts equal to one (k=1).
7. Consider Q as a draft of $F_{k+\text{1}}$.
8. Subtract one unit from $n_{q}$ for each member of $F_{k}$ such as p and for each member of $S_{P}$such as q (all $q_{s}$that are defeated by p).
9. If $n_{q}=\text{0}$, add q to Q.
10. If Q is empty, the sorting process is over.
11. If Q is not empty, consider $F_{k+\text{1}}$ equal to one.
12. Add one unit to k and go to Step 7.

**Fig 11:**
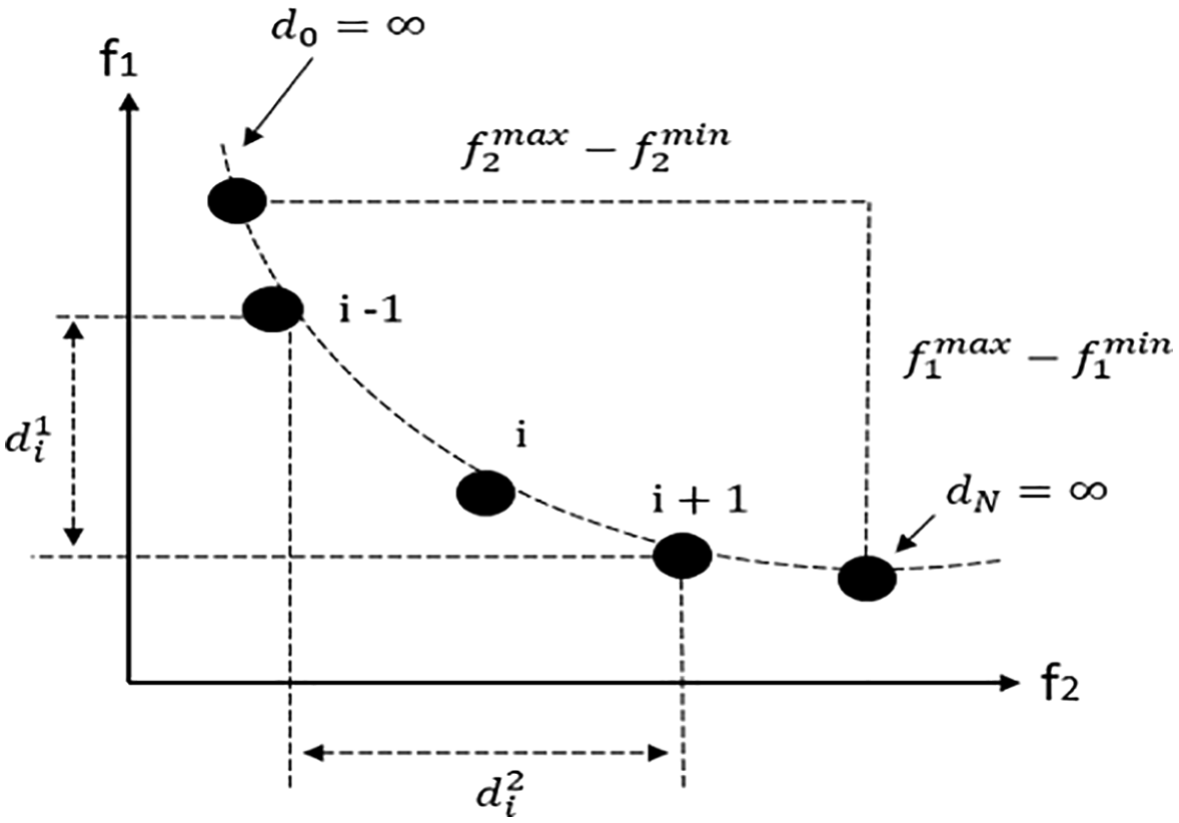
A graphical illustration of crowding distance.


Following that, crowding distance is used, which refers to the ratio of the distance between the preceding and succeeding solutions of a particular member in the Pareto front to the difference between the maximum and minimum values among the Pareto front members, and is calculated using expression (61), and is shown graphically in Fig 11.

$$\begin{array}{c} \overset{j}{\underset{i}{d}}=|\frac{\overset{before}{\underset{j}{f}}-\overset{next}{\underset{j}{f}}}{\overset{max}{\underset{j}{f}}-\overset{min}{\underset{j}{f}}}| \end{array}$$ (61)

The crowding distance of the whole Pareto-front with the number of m members is equal to the sum of the crowding distance of each member following equatain (62).


$$\begin{array}{c} d_{i}=\overset{\text{1}}{\underset{i}{d}}+\ldots +\overset{m}{\underset{i}{d}}=\sum_{j=\text{1}}^{m}\overset{j}{\underset{i}{d}} \end{array}$$ (62)

Therefore, the NSGA-II algorithm steps for selecting a method and forming a new population (New Population Selection) are as follows:

1. Two members of the population are randomly selected.
2. If the rank of the two selected members is different, the member with the lower rank is selected.
3. Otherwise, the member with the largest crowding distance is selected.

## Numerical experiments

The model is first evaluated using a small-scale case study to validate the proposed approach and examine its correctness and behavior under controlled conditions. This validation problem is solved using the LP-Metric method in GAMS software with the CPLEX solver (version win64 25.1.2, running on a PC with an 11th Gen Intel® Core™ i7-1165G7, 2.80GHz CPU, and 8 GB RAM). The case study is based on data from Tehran’s District 22, Iran, where the depot is located adjacent to Treata Hospital as part of a medication distribution initiative for the district. The small-scale instance comprises two AMLs, five normal patients (numbered from 1 to 5), and three emergency patients (numbered from 6 to 8), all geographically distributed across District 22. Following the validation phase, larger problem instances derived from a real pharmaceutical distribution company are considered to assess the model’s performance at a realistic scale, for which the NSGA-II metaheuristic algorithm is applied using MATLAB software (version R2020a). The geographic coordinates of all nodes in the small-scale case study are summarized in Table 3 for reproducibility purposes.

**Table 3:** Geographic coordinates of nodes in the small-scale case study (Tehran’s District 22).

Node	Type	Latitude	Longitude
Depot	Depot (Treata Hospital area)	35.757728	51.224172
AML 1	Automated Medicine Locker	35.754175	51.260350
AML 2	Automated Medicine Locker	35.729444	51.246613
Patient 1	Normal	35.761188	51.199744
Patient 2	Normal (assigned to AML 1)	35.762320	51.244907
Patient 3	Normal (assigned to AML 1)	35.748877	51.241045
Patient 4	Normal	35.750971	51.177556
Patient 5	Normal	35.762567	51.169477
Patient 6	Emergency	35.744355	51.228421
Patient 7	Emergency (assigned to AML 2)	35.722824	51.257943
Patient 8	Emergency	35.747112	51.154568

This problem is raised in two different scenarios. In the first scenario, the routes of patient and AML delivery operations are different, and a vehicle distributing medicine among patients is not allowed to travel to an AML. In the second scenario, there is no distinction between vehicles. To avoid prolonging the execution time, only three speed levels, 30, 40, and 50 km/h, were considered. Also, the service time to each AML is 7 minutes, and the service time to each patient is estimated to be 15 minutes. The normal radius of the first AML is 0.75 km, and the second AML is 2 km. Also, the cost of tolls is randomly calculated between 1 and 4 currency units between routes. The cost of the driver’s wage is 25 currency units per hour during normal hours and 35 currency units during irregular hours, and $\left[t_{w},t_{a}\right]=[\text{8},\text{1}\text{8}]$. The set of vehicles is k={1,2,3} and m={1}.

The results of both scenarios are presented twice: once by considering only the first objective function (minimizing economic costs), and once by considering all three objective functions simultaneously using the LP-metric method.

**First scenario – minimizing the first objective function only (Z₁):** After solving the problem, both AMLs are selected for replenishment. Specifically, normal patients 2 and 3 are allocated to AML 1, while emergency patient 7 is allocated to AML 2. The remaining patients are served through home delivery via three vehicle routes originating from the depot. Vehicle 1 travels the route: Depot → Patient 5; Vehicle 2 travels: Depot → Patient 6 → Patient 1 → Patient 4; and Vehicle 3 travels: Depot → Patient 8. In addition, the AML resupply vehicle follows the route: Depot → AML 2 → AML 1. When only the economic dimension is considered, all vehicles utilize type 3 fuel, which carries the lowest unit cost regardless of its pollution level. Furthermore, to minimize driver wage costs, all patient-serving vehicles operate at speed level 3 (50 km/h), i.e., the fastest available speed, thereby reducing total travel time. The numerical outputs of this scenario are reported in Table 4.

**Table 4:** The outputs of the first scenario, only optimizing the first objective function.

Variables		Values
Arrival time	${\text{t}}_{\text{53}}$	723.602
	${\text{t}}_{\text{81}}$	720
	${{\text{t}}^{\prime }}_{\text{11}}$	709.203
	${\text{t}}_{\text{12}}$	711.687
	${{\text{t}}^{\prime }}_{\text{21}}$	699.486
	${\text{t}}_{\text{42}}$	729.071
	${\text{t}}_{\text{62}}$	692
Vehicle departure time (minutes)	$\overset{\text{3}}{\underset{\text{o}}{\lambda }}$	700
	$\overset{\text{1}}{\underset{\text{o}}{\lambda }}$	699.004
	$\overset{\text{1}}{\underset{\text{o}}{\lambda^{\prime }}}$	664.558
	$\overset{\text{2}}{\underset{\text{o}}{\lambda }}$	661.916
Emergency coverage radius (km)	$\overset{\text{e}}{\underset{\text{2}}{\text{r}}}$	1.179
Objective function	${\text{Z}}_{\text{1}}$	97.884
	${\text{Z}}_{\text{2}}$	0.054
	${\text{Z}}_{\text{3}}$	0.9054

**First scenario – considering all three objective functions (LP-metric, w_1_ = 0.8, w_2_ = w_3_ = 0.1):** The LP-metric approach was applied to solve this instance with weights of 0.8 for the first objective function (costs) and 0.1 each for the second (GHG emissions) and third (patient satisfaction). The AML allocation remains identical to the previous case: normal patients 2 and 3 are assigned to AML 1, and emergency patient 7 is assigned to AML 2. However, incorporating the emission and satisfaction objectives leads to changes in two of the vehicle routes. Specifically, compared to the cost-only solution, Vehicle 2’s route changes from Depot → Patient 6 → Patient 1 → Patient 4 to Depot → Patient 4 only, while Vehicle 1’s route changes from Depot → Patient 5 to Depot → Patient 5 → Patient 1 → Patient 6. The remaining routes stay the same. This routing adjustment is driven by the time window constraint of the first emergency patient, which makes selecting the minimum-cost route infeasible when patient satisfaction is simultaneously optimized. In this case, all vehicles switch to type 1 fuel, which has the lowest emission factor but is slightly more expensive, reflecting the incorporation of the environmental objective. Speed level 3 remains the selected speed for all patient vehicles. The emergency coverage radius for AML 2 is determined to be 1.179 km. The detailed numerical outputs are reported in Table 5.

**Table 5:** The outputs of the first scenario with the LP-metric method.

Variables		Values
Arrival time	$t_{\text{4}\text{3}}$	724.142
	$t_{\text{5}\text{1}}$	681.957
	${t^{\prime }}_{\text{1}\text{1}}$	798
	$t_{\text{1}\text{1}}$	700.313
	${t^{\prime }}_{\text{2}\text{1}}$	717.377
	$t_{\text{6}\text{1}}$	720
	$t_{\text{8}\text{2}}$	720.996
Vehicle departure time (minutes)	$\overset{\text{3}}{\underset{o}{\lambda }}$	700
	$\overset{\text{1}}{\underset{o}{\lambda }}$	657.957
	$\overset{\text{1}}{\underset{o}{\lambda^{\prime }}}$	677.845
	$\overset{\text{2}}{\underset{o}{\lambda }}$	700
Vehicle finish time (minutes)	$\overset{\text{3}}{\underset{n+\text{1}}{\lambda }}$	735
	$\overset{\text{1}}{\underset{n+\text{1}}{\lambda }}$	735
	$\overset{\text{1}}{\underset{n+\text{1}}{\lambda^{\prime }}}$	735.996
	$\overset{\text{2}}{\underset{n+\text{1}}{\lambda }}$	735.996
Emergency coverage radius (km)	$\overset{e}{\underset{\text{2}}{r}}$	1.179
Objective function	$Z_{\text{1}}$	103.3035
	$Z_{\text{2}}$	0.0005425257
	$Z_{\text{3}}$	2.921

The results are summarized in Table 6 for comparison results of optimizing each objective function separately and after implementing the LP-metric. In the case that only the second objective function (minimizing greenhouse emissions) is considered to optimize, only AML 2 is selected, and patients 3 and 7 are allocated to it. The fuel used is type 1 with the minimum amount of pollution, and the level 3 speed has not been selected. If only the third objective function is considered, the problem will not be limited to the type of fuel consumed and the speed and time of the vehicle reaching the points. It only adjusts the speed within the time window and increases the patient’s satisfaction. Also, in this case, only AML 2 is selected, and patient 3 is allocated to it. Other patients are served directly. It should be noted that the total value of patients’ satisfaction is the sum of the values between 0 and 1, and as a result, it can exceed one.

**Table 6:** The summary of solutions for the first scenario.

Objective functions	Z1$({𝐰}_{1}=1)$	Z2$({𝐰}_{2}=1)$	Z3$({𝐰}_{3}=1)$	LP-metric (${𝐰}_{1}=0.8,{𝐰}_{2}=0.1)$
First objective (costs)	97.884	202.287	227.552	103.3035
Second objective (emissions)	0.054	0.000497671	0.008	0.0005425257
Third objective (patient satisfaction)	0.9054	3.535	6.8818	2.921

**Second scenario – minimizing the first objective function only (Z**_**1**_**):** For the second scenario, the set of vehicles is k={1,2,3,4}. Other assumptions are the same as in the first scenario. After solving the problem, only AML 2 is selected for replenishment, and patients 3 and 7 are allocated to it. The remaining patients are served through home delivery via four vehicle routes originating from the depot. Specifically, Vehicle 1 travels the route: Depot → Patient 5; Vehicle 2 travels: Depot → Patient 4 → Patient 1; Vehicle 3 travels: Depot → Patient 8; and Vehicle 4 travels: Depot → AML 2 → Patient 6. All vehicles travel at speed level 3 (50 km/h), i.e., the highest available speed, and utilize type 3 fuel, which has the lowest cost among all fuel types. Compared to the first scenario, by spending marginally more resources in this configuration, the satisfaction level of patients has increased from 2.846 to 4.761, reflecting the operational flexibility gained when all vehicles can serve both patients and AMLs without route-type restrictions.

**Second scenario – considering all three objective functions (LP-metric, w**_**1**_ **= 0.8, w**_**2**_ **= w**_**3**_ **= 0.1):** The AML selection and patient allocation remain unchanged: only AML 2 is opened, with patients 3 and 7 assigned to it. However, incorporating GHG emissions and patient satisfaction alongside cost leads to notable changes in the routing structure. In this case, all patients are served through five separate routes: Vehicle 1 travels: Depot → Patient 5; Vehicle 2 travels: Depot → Patient 4; Vehicle 3 travels: Depot → Patient 8; Vehicle 4 travels: Depot → Patient 1 → Patient 6 → AML 2 → Patient 2. Compared to the cost-only solution, the most significant change is that Vehicle 4 now consolidates multiple visits including AML 2 replenishment into a single route that also serves Patients 1, 6, and 2, which reflects the multi-objective trade-off between cost efficiency, emission reduction, and time-window compliance for patient satisfaction. The detailed numerical outputs are reported in Table 7. In this scenario, if only the third objective function is optimized, no AML is opened, and all patients are served directly from the depot. If only the second objective function is considered for optimization, AML 1 is selected, and patients 2 and 3 are allocated to it.

**Table 7:** The summary of solutions for the second scenario.

Objective functions	${𝐙}_{1}(w_{1}=1)$	${𝐙}_{2}(w_{2}=1)$	${𝐙}_{3}(w_{3}=1)$	$𝐋𝐏-𝐦𝐞𝐭𝐫𝐢𝐜(w_{1}=0.8,w_{2}=0.1)$
First objective (costs)	94.056	192.807	185.06	102.684
Second objective (emissions)	0.043	0.00035491956	0.064	0.00041224146
Third objective (patient satisfaction)	2.846	4.023	7.994	4.761

### Solving a case study with the NSGA-II method

**Fig 12:**
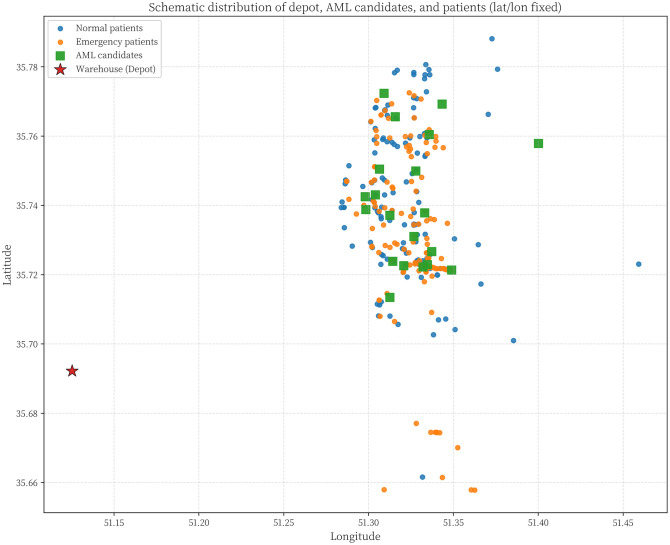
Schematic geographic distribution of the depot, patients, and candidate AML locations.

In this section, the model is implemented on a realistic scale. The Tehran branch of the mentioned company includes two parts of East and West Tehran, with a separate transport fleet for each region. This plan has been developed for the daily distribution in West Tehran, which is managed directly from the central warehouse located in Qods, with coordinates (Latitude: 35.6922, Longitude: 51.1253). The geographic distribution of the depot, AML candidate locations, and patients across West Tehran is illustrated in Fig 12, which presents a schematic map generated from WGS84 coordinate data. Four vehicles are allocated for the distribution plan in West Tehran, and the company’s transport fleet is equipped with intelligent tracking systems; drivers are not required to return to the depot after completing their routes.

Patients who do not require immediate medication are considered second priority and categorized as normal patients, while those requiring urgent delivery are classified as emergency patients. There are 20 candidate AML locations available across West Tehran, and the objective is to determine which AMLs should be replenished based on the geographic distribution of patients. The study involves a total of 280 patients, of whom 136 are in emergency condition and the remaining are classified as normal patients. As shown in Fig 12, both patient groups and AML candidates are geographically concentrated in the central and eastern parts of West Tehran, while the depot is situated to the west, reflecting a realistic last-mile distribution scenario.

**Fig 13:**
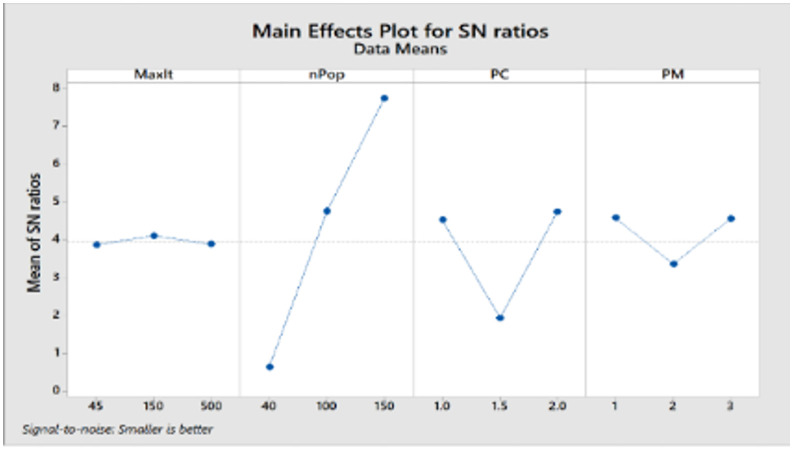
Outputs of the Minitab for parameter tuning by the Taguchi method.

In the presented problem, the normal coverage radius is set to 2.5 km. The loading time for each vehicle is assumed to be 45 minutes, and 12 speed levels ranging from 10 to 120 km/h are defined. Other inputs to this problem are the same as in the case solved by LP metrics. For parameter tuning of the algorithm, which includes the number of iterations and population size, and the crossover and mutation percentage, the optimal values were extracted using the Taguchi method with the help of Minitab software, and the output is shown in Fig 13.

That means, for the problem with defined dimensions, the number of iterations (MaxIt) is equal to 150, and the population size (nPop) is 150. According to the slope of the drawn line, the larger the population, the higher the quality of the answer. Our chosen nPop is 150 according to the available computational power of the system, considering the high dimensions of the problem and the speed of its execution. The crossover probability (PC) is 2, and the mutation probability (PM) is 1. In summary, the standardized values are presented in Table 8.

**Table 8:** Signal-to-noise ratios.

Level	MaxIt	nPop	PC	PM
1	3.8752	0.6476	4.5301	4.5817
2	4.1125	4.7674	1.9429	3.3651
3	3.8887	7.7478	4.7548	4.5662
Delta	0.2373	7.1002	2.8119	1.2166
Rank	4	1	2	3

According to the Rank row, population size has the most influence, while the number of iterations has the least.

#### Results of computational experiments.

**Fig 14:**
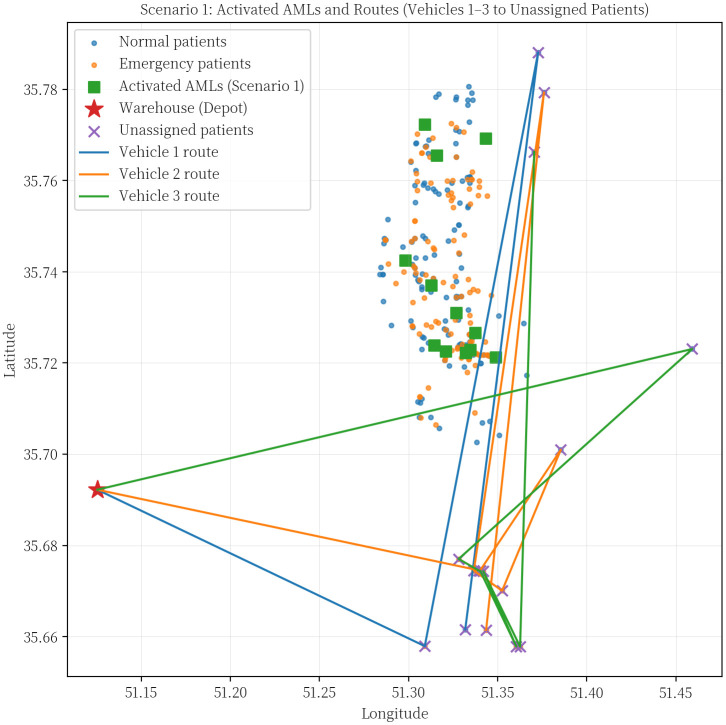
Activated AMLs and routes (vehicle 1-3 to unassigned patients).

In the first scenario, one vehicle was allocated for traveling to the AMLs, while the remaining three vehicles were responsible for delivering medicine directly to patients. The first Pareto front, which is a collection of the best answers, has 56 members after the problem has been solved. Several solutions with different emergency coverage radii were presented to compare the results. Vehicles 1–3 are related to home delivery, and vehicle 4 travels to AMLs. The routes of which are shown in Fig 14.

**Table:** 

Vehicle 1:	[1,3,11,13]
Vehicle 2:	[1,2,5,12,29,30,32]
Vehicle 3:	[1,4,10,14,26,28,31,33,34]
Vehicle 4:	[1,35,38–40,42–46,48,51,52]

The program has made an effort to distribute the majority of patients who are situated in this area by opening the AMLs in the middle of the map. To avoid further complexity, we have displayed these areas only as activated AMLs and patients within the corresponding coverage radius. Furthermore, routes of unassigned patients who are served by home delivery services are shown as colored lines.Table 9 presents the results of the first scenario, while Table 10 displays the outcomes related to the fifth member of the Pareto front.

**Table 9:** The output of the first scenario.

Variables		Values
Emergency coverage radius (km)	$\overset{e}{\underset{i}{r}}$	1.4377
Vehicle departure time (minute)	$\overset{\text{1}}{\underset{o}{\lambda }}$	536
	$\overset{\text{2}}{\underset{o}{\lambda }}$	541
	$\overset{\text{3}}{\underset{o}{\lambda }}$	535
	$\overset{\text{4}}{\underset{o}{\lambda }}$	535
Vehicle finish time (minute)	$\overset{\text{1}}{\underset{n+\text{1}}{\lambda }}$	931.14
	$\overset{\text{2}}{\underset{n+\text{1}}{\lambda }}$	629.57
	$\overset{\text{3}}{\underset{n+\text{1}}{\lambda }}$	633.38
	$\overset{\text{4}}{\underset{n+\text{1}}{\lambda }}$	756.14
Objective functions	$Z_{\text{1}}$	414.5012
	$Z_{\text{2}}$	0.0016538977
	$Z_{\text{3}}$	7.3958

**Table 10:** The fifth member of Pareto front.

Objective functions and values	
${\text{Z}}_{\text{1}}$	244.7093
${\text{Z}}_{\text{2}}$	0.0018074815
${\text{Z}}_{\text{3}}$	4

According to Tables 9 and 10, we observe that in the fifth member, with the increase in the emergency radius, a smaller number of AMLs have been used for refilling, which has caused a decrease in the satisfaction level of patients. However, we had a significant reduction in the cost of transportation (approximately 170,000 Tomans). In the first member, all vehicles use fuel type 1. According to the type of fuel selection, the amount of GHG emissions has increased slightly in the fifth member, which can be said to be due to the model’s priority in saving economic costs, including fuel costs.

In the second scenario, as shown in Table 11 to make a better comparison of the results, we chose the first two members of the front, which have an infinite crowding distance. That means these members are the first and last members of the Pareto front. Compared to the previous scenario, fewer AMLs are used in the second scenario. In this member, 10 out of 20 candidate AMLs are selected for refilling.

**Table 11:** The objective function values of the second scenario.

	Objective functions	Values
Member 1	$Z_{\text{1}}$	407.2993
	$Z_{\text{2}}$	0.0015121420
	$Z_{\text{3}}$	8.3941
Member 2	$Z_{\text{1}}$	250.2560
	$Z_{\text{2}}$	0.0020332491
	$Z_{\text{3}}$	4.6030

**Table:** 

Vehicle 1:	35
Vehicle 2:	[5,12,26,28,29]
Vehicle 3:	[2–4,11,13,14,30,31,33,37,39,44,46,49,51–53]
Vehicle 4:	[10,32,34,45]

In the second scenario, by comparing the objective functions of the two proposed members, it was observed that in the second member, costs were reduced by slightly lowering the level of patient satisfaction, decreasing the emergency coverage radius, and adding one patient to the delivery tour. The number of selected AMLs has remained constant. In the second member, the level of GHG emissions has increased. The trade-off is between choosing fewer GHG emissions and more patient satisfaction at a higher cost versus more GHG emissions and less patient satisfaction at a lower cost.

### Investigating the efficiency of the metaheuristic method

By solving the problem across different dimensions, the efficiency of the two applied methods, the LP-metric method and the NSGA-II algorithm, was investigated. So, to examine Pareto-fronts’ efficiency, the mean ideal distance (MID) and diversity comparison have been calculated, which are explained in the following. In Table 12, the dimensions of the problems solved by the NSGA-II algorithm have been demonstrated, and the results are given in Table 13.

**Table 12:** Dimensions of problems solved by the algorithm.

Problem	AMLs number	Patients number	Active AMLs	Tour length
1	2	5	1	4
2	5	12	1	5
3	4	21	3	6
4	2	8	1	6
5	4	24	4	7
6	3	10	1	7
7	3	15	2	8
8	4	16	3	9
9	3	12	1	9
10	5	33	4	13
11	5	27	5	15
12	5	33	5	25
13	5	33	5	29
14	6	33	5	32
16	15	33	7	33
15	10	33	6	33
17	20	46	9	35
18	20	56	10	41
19	20	66	13	48

**Table 13:** The output of the problem in different dimensions with the two mentioned methods.

	NSGA-II				LP-metric				Comparison		
Problem	Z1	Z2	Z3	Runtime (s)	Z1	Z2	Z3	Runtime (s)	$\Delta_{1}$	$\Delta_{2}$	$\Delta_{3}$
1	41.4495962	0.0005770	0.0000000	355.939	41.242	0.000842	0.0000	1.197	0.00503361	0.3144823	0
2	58.6144122	0.0013325	0.0906983	442.961	57.494	0.000900	1.0000	116.198	0.01948746	0.48055892	0.90930175
3	64.1494704	0.0028725	0.1791200	1735.36	64.125	0.006109	0.8280	1768.699	0.0003816	0.52976477	0.7836715
4	78.9269947	0.0010198	0.6724104	433.119	75.3036	0.005825	0.3885	949.884	0.04811715	0.82491055	0.73078619
5	81.4760507	0.0130112	1.3280700	1961.4	76.394	0.007168	1.8240	3087.123	0.06652421	0.81511077	0.27189145
6	90.3000109	0.0003540	0.2652110	533.154	78.846	0.005777	1.2410	1713.647	0.14527067	0.93871521	0.78628592
7	96.2105244	0.0006000	1.5081042	655.196	82.758	0.007945	2.8450	7226.561	0.16255256	0.92448127	0.46991065
8	98.2910403	0.0005434	1.3698133	735.166	97.963	0.004651	2.0000	7204.277	0.00334861	0.88316956	0.31509333
9	116.6464870	0.0007440	2.7172680	591.384	97.336	0.005708	2.6087	7635.117	0.19838998	0.86964138	0.04161765
10	117.6770000	0.0006917	1.5251899	1942.05	101.232	0.004788	1.9310	10802.81	0.16244634	0.85552939	0.21013903
11	130.4460395	0.0003199	2.1280203	1827.315							
12	274.6241000	0.0015031	4.6713900	1909.15							
13	302.8116500	0.0018355	5.5702400	1941.25							
14	345.3791000	0.0007019	5.8718500	2187.173							
16	342.0139000	0.0021398	5.4229700	2882.827							
15	351.4616500	0.0005597	5.5524240	2361.5							
17	383.6842000	0.0007330	7.5738469	3683.51							
18	402.7284000	0.0092513	5.4134808	8600.915							
19	494.6318660	0.0009840	7.1303140	10107.5							

As shown in Table 13, the NSGA-II algorithm consistently solves problems in significantly less time than the LP-metric method. For instance, for Problem 10 (5 AMLs, 33 patients), NSGA-II required 1,942 seconds, whereas the LP-metric method required 10,803 seconds. From Problem 10 onward, the LP-metric method exceeded the 3-hour time limit in GAMS, making it impractical for larger instances. This confirms that the NSGA-II algorithm is the preferred approach for realistic-scale problems, offering near-optimal solutions (with average deviations below 15% for z1) in a fraction of the time.

For more, the best values obtained for the objective functions are reported by the LP-metric and NSGA-II algorithms, and the results indicate a small difference between the exact and metaheuristic solutions. Based on the results, it can be concluded that the LP-metric method is more efficient than the NSGA-II algorithm only for small dimensions, and the solving problem speed for this exact method decreases with increasing the dimension problem. The last two columns of this table show the difference between the answers obtained from the expression (63). In this relation, Δi is the difference between the solutions of objective function i in exact and metaheuristic solution, $\overset{H}{\underset{i}{Z}}$ is the best value of objective function *i* in the NSGA-II algorithm and $\overset{E}{\underset{i}{Z}}$is the best value of objective function i in the LP-metric method.


$$\begin{array}{c} \Delta i=|\frac{\overset{H}{\underset{i}{Z}}-\overset{E}{\underset{i}{Z}}}{\overset{E}{\underset{i}{Z}}}| \end{array}$$ (63)

To evaluate the efficiency of the generated Pareto front, two indicators were considered: Mean Ideal Distance (MID) and Diversity Metric (DM), which are briefly described below. Then, the two indicators were measured in order of increasing problem dimension for the selected problems and were illustrated in the graph. MID is a straightforward metric that measures the average distances from an ideal point. It is calculated through expression (64). A lower value of MID indicates a better performance of the algorithm.


$$\begin{array}{c} MID=\frac{\sum_{i=\text{1}}^{n}\sum_{m=\text{1}}^{M}{\left(\frac{f_{mi-\overset{best}{\underset{m}{f}}}}{\overset{max}{\underset{mtotal}{f}}-\overset{min}{\underset{mtotal}{f}}}\right)}^{\text{2}}}{n} \end{array}$$ (64)

M indicates the $m^{th}$ objective function and M is the number of objectives, which in our problem, equals to 3 and n is the number of Pareto members. $\overset{max}{\underset{mtotal}{f}}$ and $\overset{min}{\underset{mtotal}{f}}$ are the maximum and minimum values of the $m^{th}$ objective function between solutions and $\overset{best}{\underset{m}{f}}$ is the ideal value for objective m.

DM shows the diversity of the Pareto solutions and is calculated through the expressions (65) and (66). The higher the value of DM, the better the algorithm’s performance.


$$\begin{array}{c} d_{i}={max}_{j}\{\sum_{m=\text{1}}^{M}{\left(\overset{m}{\underset{i}{f}}-\overset{m}{\underset{j}{f}}\right)}^{\text{2}}\} \end{array}$$ (65)

$$\begin{array}{c} DM=\sum_{i=\text{1}}^{n}d_{i} \end{array}$$ (66)

In above, $\overset{m}{\underset{i}{f}}$ and $\overset{m}{\underset{j}{f}}$ are the values of the objective function m of the two Pareto solutions i and j*.* Table 14 shows the MID, and DM values for problems 1–10.

**Table 14:** Performance of NSGA-II in terms of DM and MID.

Problem#	𝐌𝐈𝐃	𝐃𝐌
1	0.260679	8.778067
2	0.130767	142.24
3	0.124822	1,020.17
4	0.732361	21.52
5	0.133956	547.29
6	0.293941	170.55
7	0.316038	146.3475
8	0.342491	122.4597
9	0.193604	453.253
10	0.136647	605.0354

**Fig 15:**
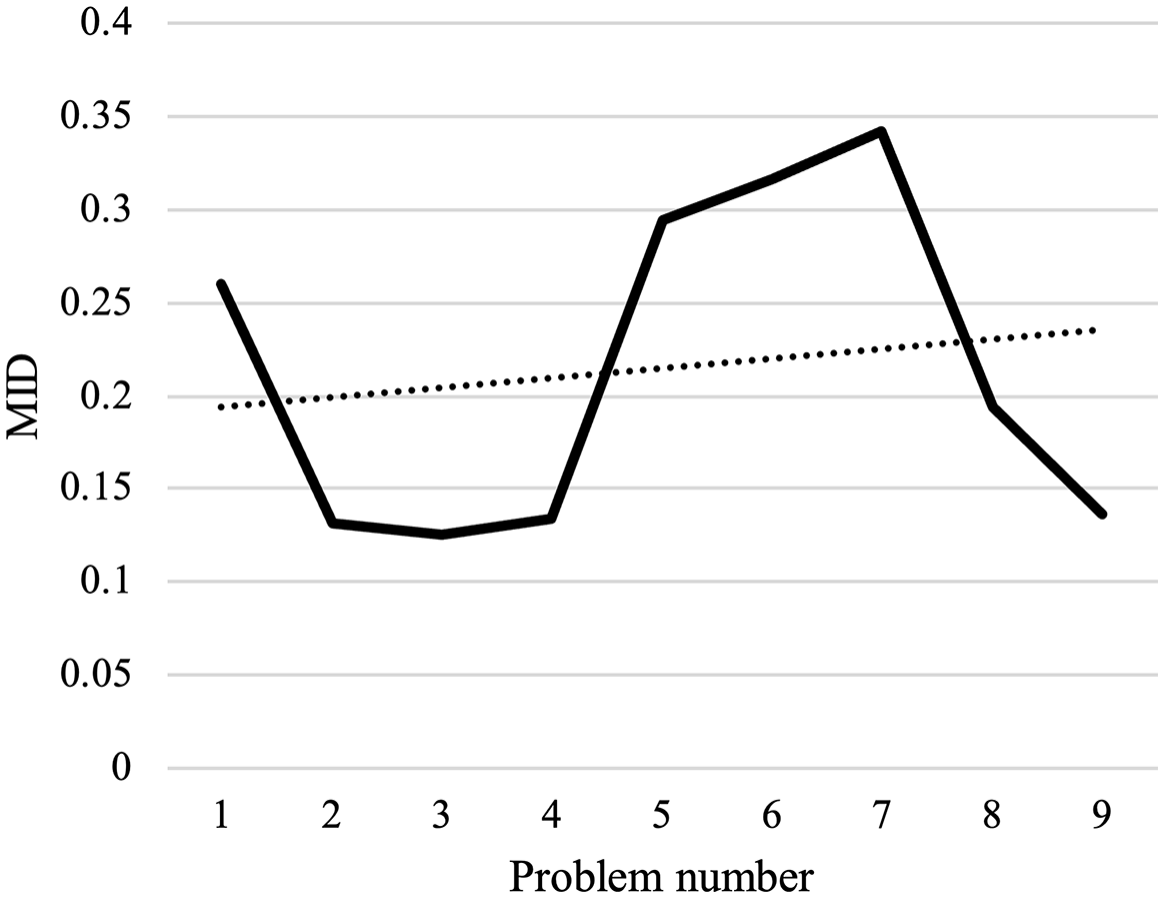
The MID values of the problems.

**Fig 16:**
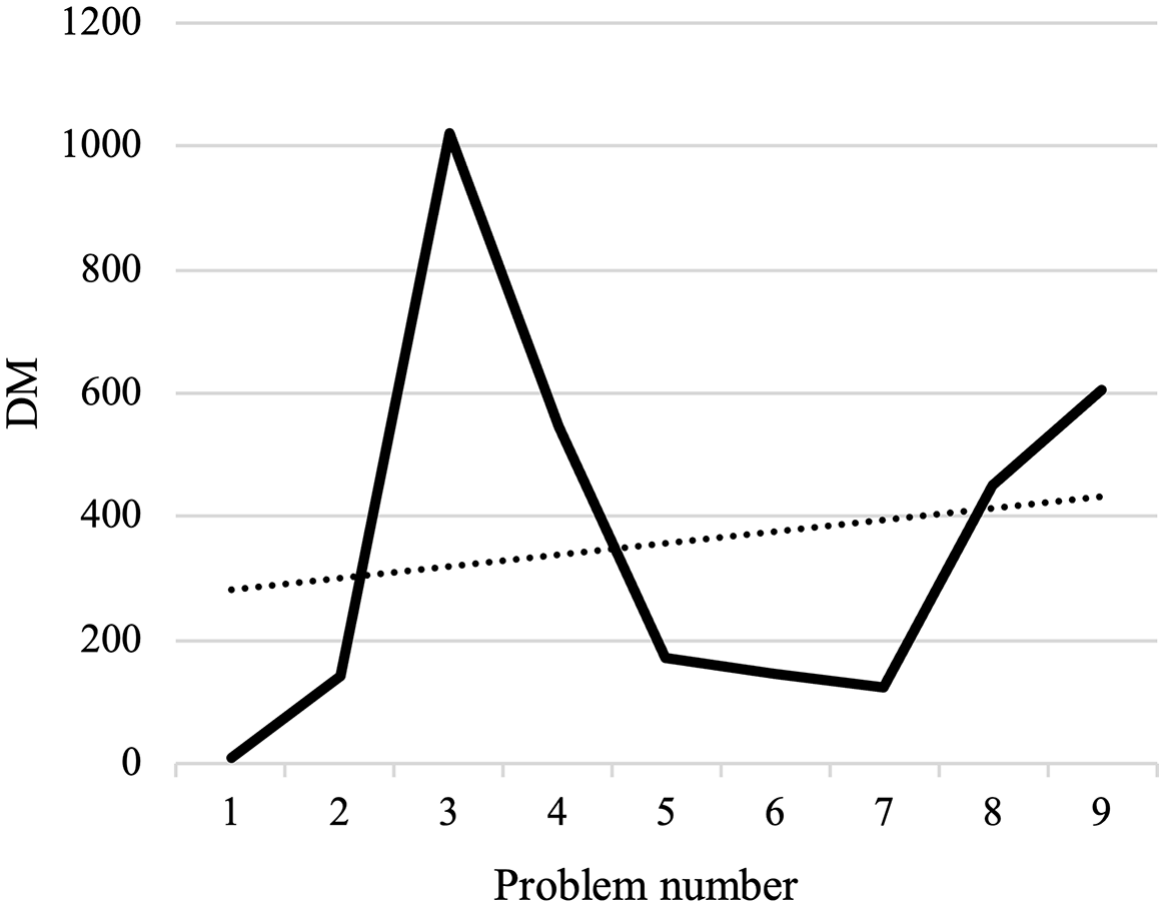
The DM values of the problems.

As shown in Figs 15 and 16, if the results for Problem 4 are disregarded, it can generally be concluded that increasing the problem dimension leads to an increase in the average distance from the ideal point, indicating that the algorithm’s efficiency does not improve with larger problem sizes. Additionally, in the low dimensions, the diversity of the Pareto solutions is not sufficient, because the solutions gather approximately at one point, and by increasing the problem dimension, this diversity improves.

## Sensitivity analysis and managerial implications

### The effect of using AML on objective functions

To analyze and estimate the use of AMLs in distribution and to determine the percentage of cost savings by using AMLs, we increased the number of AMLs from zero to 3, assuming a constant number of patients (8 people). The results are shown in Table 15 for both scenarios.

**Table 15:** The results of both scenarios with the addition of an AML on the small-scale cases.

	Number of AMLs	$𝐙1(w_{1}=1)$	𝐙2(×1000)	𝐙3	Percentage of savings
First scenario	0	136.428	72	5.808	–
	1	131.007	70	4.999	3.97352
	2	127.904	63	2	2.36858
	3	88.322	60	1.615	30.9466
Second scenario	0	123.254	64	5.945	–
	1	116.41	86	5.24	5.55276
	2	111.277	103	2.07	4.40941
	3	79.928	61	2	28.172

**Fig 17:**
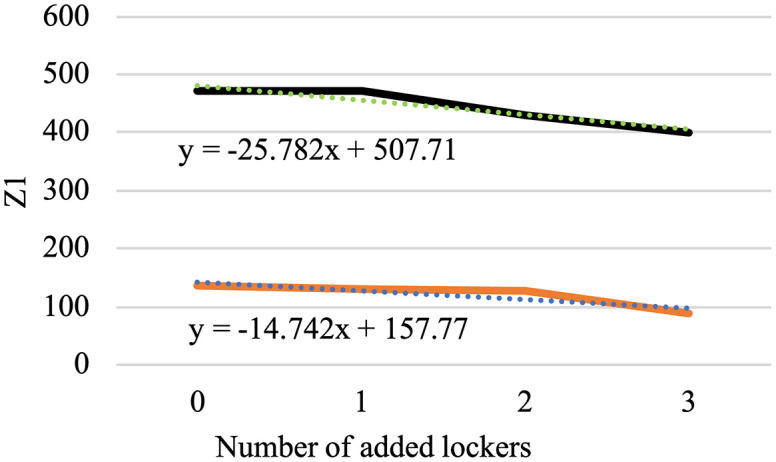
The comparison of the results of Z1 of two samples for the first scenario, with adding an AML.

**Fig 18:**
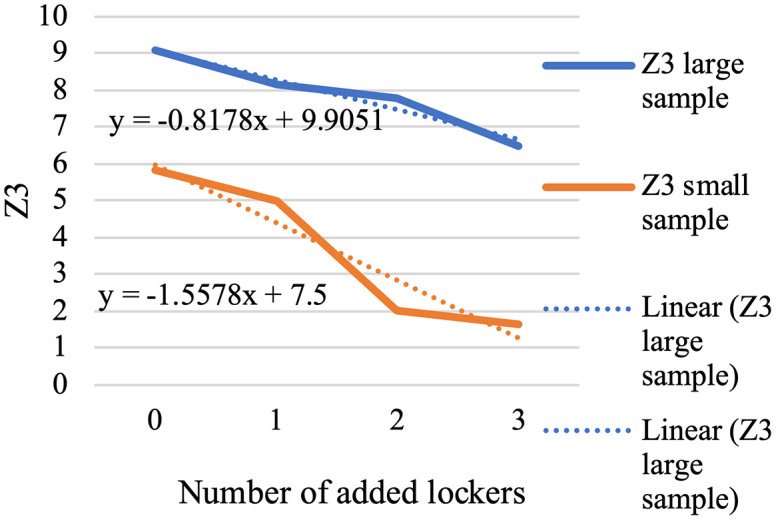
The comparison of the results of Z3 of two samples for the first scenario, with the addition of an AML.

Furthermore, the problem was solved on a larger scale, assuming 65 patients and using between 8–11 AMLs for the first scenario. The results are presented in Table 16. The results of both samples for the first and third objective functions are given in Figs 17 and 18. As the graphs show, the trend of cost reduction and patient satisfaction is similar for both large and small scales with the increase in the number of AMLs. But the amount of this reduction on a large scale for costs is an average of 25.782%. Therefore, according to the scale of the problem, it is recommended to use more AMLs on large-scale cases. In small-scale cases, since the level of patient satisfaction decreases more intensely, more use of AMLs depends on the policy of the distribution company.

**Table 16:** The results of the first scenario with the addition of an AML on large-scale cases.

Number of AMLs	Z1	Z2	Z3	Percentage of savings
8	471.7178	0.0037	9.06	–
9	472.1512	0.0005	8.1541	−0.091877
10	428.9602	0.02347	7.7636	9.1477
11	400.1761	0.00165	6.464	6.71021

**Fig 19:**
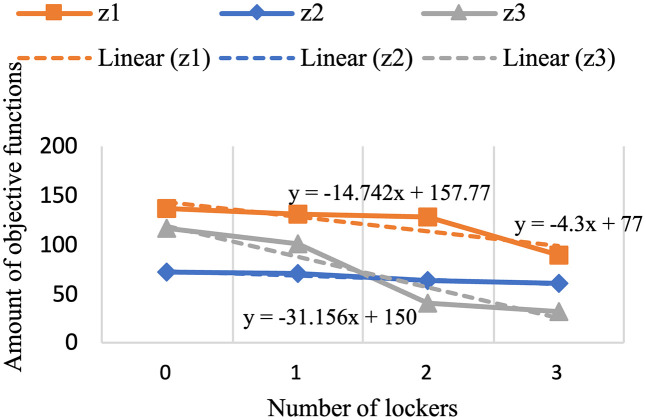
The results of the first scenario with the addition of an AML.

**Fig 20:**
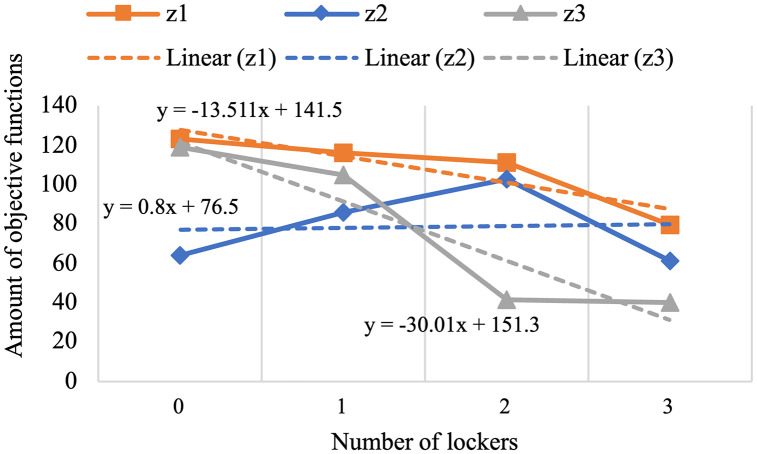
The results of the second scenario with the addition of an AML.

As shown in Figs 19 and 20, for both scenarios, the amount of costs is reduced by increasing the number of AMLs. Also, the use of AMLs in both scenarios will have an opposite effect on the level of patient satisfaction (patients not allocated to AMLs), and part of this decrease can be due to the decrease in the number of patients who are served directly from the depot. By increasing the number of AMLs, the amount of pollution and GHG emissions decrease (For better presentation, the values of the third objective function have been multiplied by 20).

As shown by the linear trend diagram of all three objective functions, and based on the linear trend equation, it was concluded that, on average, increasing the number of selected AMLs by one could result in a 14.742% cost saving. Additionally, adding one AML can reduce GHG emissions by 4.3% on average.

In the second scenario, with the increase in the number of AMLs, the transportation costs with a lower intensity (13.511%) have been reduced compared to the first scenario. The decreasing trend of patient satisfaction is similar to the first scenario, with a lower slope. But the GHG emissions in this scenario have had an ascending trend on average.

### Analysis of the relation between the two scenarios

**Fig 21:**
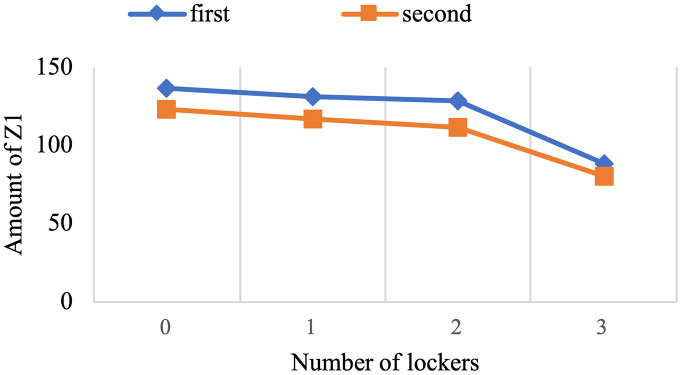
The first objective function of both scenarios with the addition of an AML.

By comparing the obtained results, as shown in Fig 21, the second scenario is always more economical than the first scenario. With the increase in the number of AMLs, it can be assumed that the decreasing trend of both scenarios is similar.

**Fig 22:**
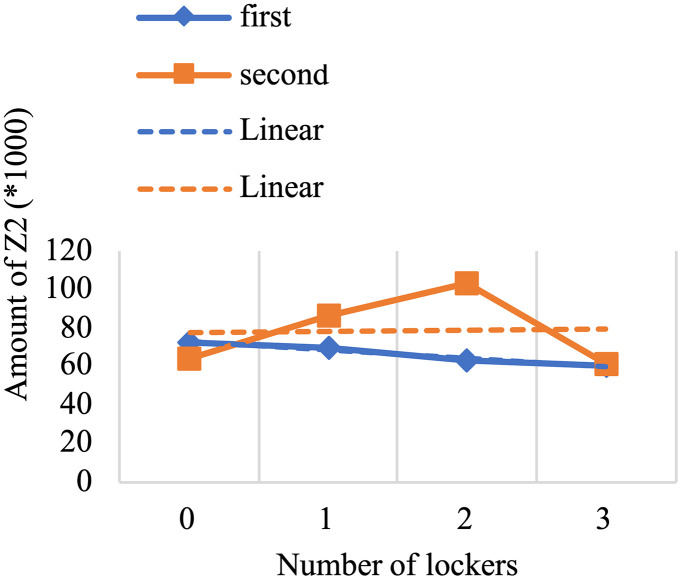
The second objective function of both scenarios with the addition of an AML.

**Fig 23:**
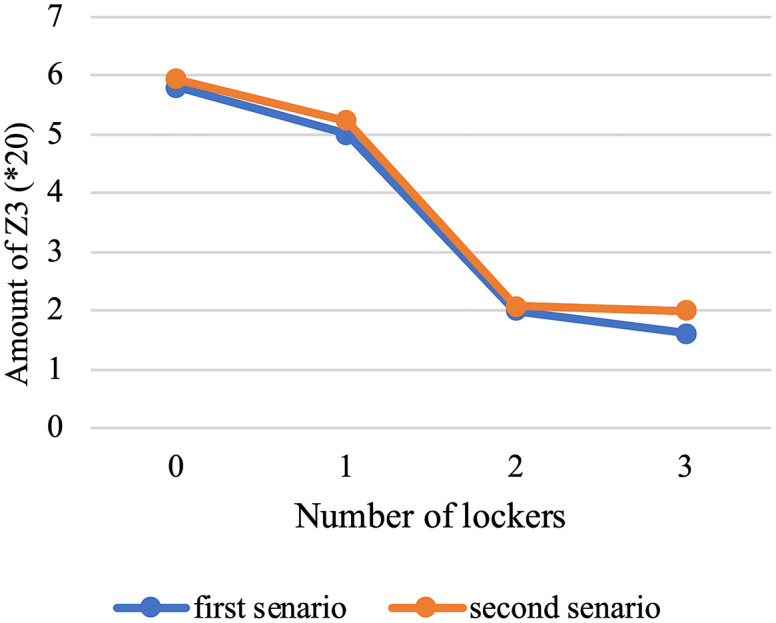
The third objective function of both scenarios with the addition of an AML.

As shown in Fig 22, the first scenario performs better in terms of GHG emissions. It should be noted that since each tour has specific tolls and the shortest path is not necessarily the best regarding emissions, the trend depicted in Fig 22 is different for the two scenarios. Because only costs are considered, choosing a longer but less expensive route in the second case is possible by selecting an AML and adjusting routing priority. However, in the case of using three AMLs, the amount of pollution emission has reached the same value for both scenarios. If three AMLs were used, the second scenario could perform better than the first scenario in terms of both economic efficiency and the level of patient satisfaction (Fig 23).

### Relation between objective functions

**Fig 24:**
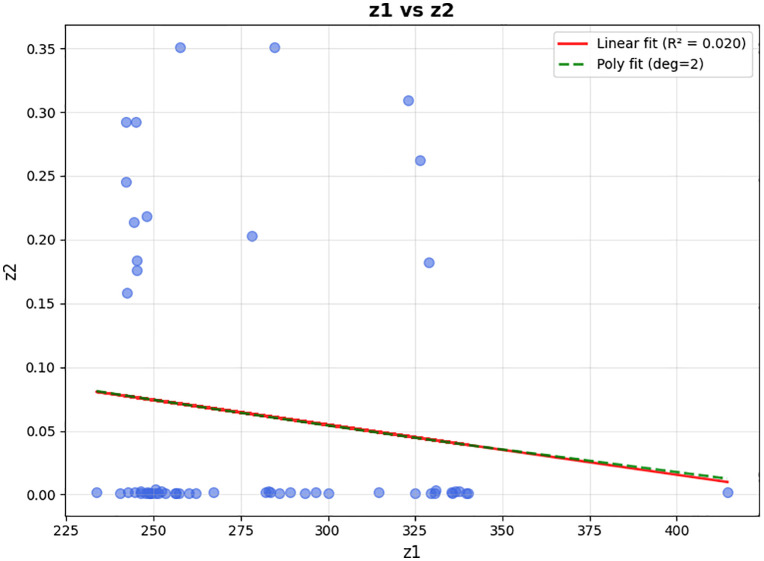
The presentation of the Pareto-front for first and second objective functions and their relationship.

In the first scenario, the algorithm tried to reduce the cost function. As Fig 24 shows, the two objective functions perform in contrast with each other. In other words, with the reduction of the cost and the consumption of inferior fuels that are less expensive, the amount of GHG emissions has increased. From the intensity of the changes, it was concluded that fuel consumption played a major role in the economic costs within the first objective function. It should be noted that with longer routes and higher fuel consumption, both the first and second objective functions increased. However, if fuel usage and cost are given more weight in the diagram, the two objective functions will have an inverse relationship. Another reason for this conflicting relation is the determined speed. As the speed increases, the amount of fuel consumption and GHG emissions increase, but on the other hand, the duration of time traveling the route, and as a result, drivers’ wages decrease.

**Fig 25:**
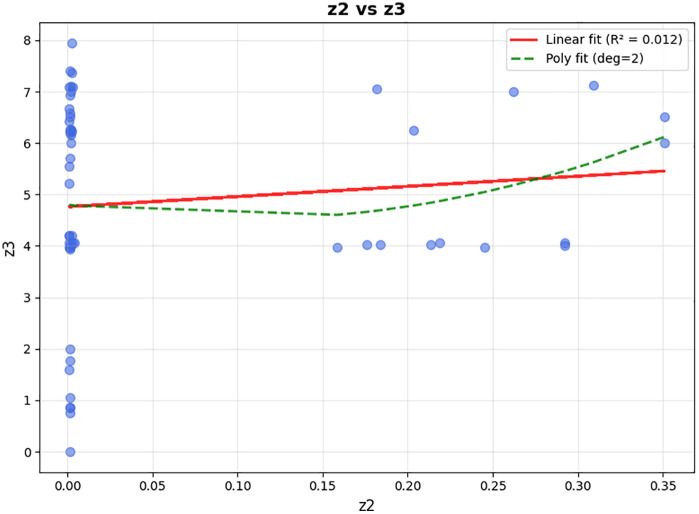
The presentation of the Pareto-front for second and third objective functions and their relationship.

As for the second and third objective functions, Fig 25 shows a direct relation between the two objective functions. In other words, as the level of patient satisfaction decreases, the second objective function decreases with a much lower slope. GHG emissions and patient satisfaction decrease whenever the speed is reduced, and the vehicle fails to visit the patients within the specified time window. In fact, more GHG emissions may cause a decrease in patient satisfaction due to more traveling nodes.

**Fig 26:**
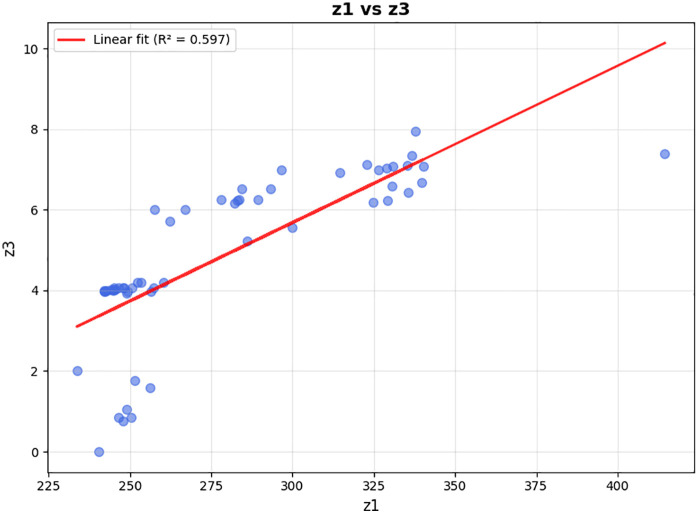
The relation between the first (Z1) and the third objective function (Z3).

Fig 26 shows the first and third objective function values of the first front as a scatter diagram. The level of patient satisfaction is dropping as costs are reduced. As a result, since the aim was to increase patient satisfaction while reducing economic expenses, the trade-off was between achieving a lower cost with a lower level of patient satisfaction and incurring a higher cost with a higher level of patient satisfaction. The steep slope of the linear fit between the data indicates that the level of patient satisfaction increases significantly with a slight increase in costs.

**Fig 27:**
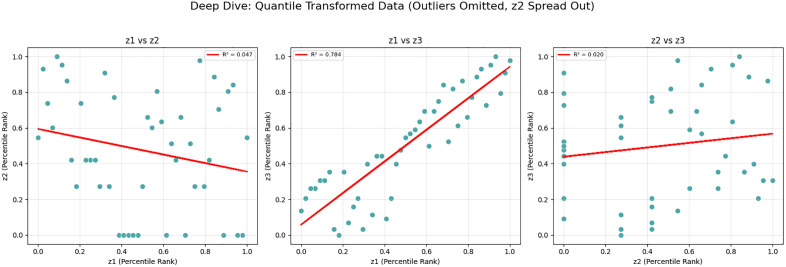
Deep dive: Quantile Transformed data (outliers omitted, Z2 spread out).

In Fig 27, we visualize the relationship between objective functions by applying a quantile transformation to the Pareto-front data. Each point represents a complex multi-dimensional trade-off rather than a simple two-dimensional functional relationship. For example, $Z_{\text{2}}$ is influenced not only by $Z_{\text{1}}$, but also by $Z_{\text{3}}$, vehicle speeds, fuel types, and tolls. This multi-factor interaction produces substantial dispersion in the Pareto-front points, which yields low MSE-based R^2^ values, indicating simple linear (or low-complexity) approximation does not capture the dependence well, consistent with strong interactions and non-linear effects across objectives and decision variables.

**Fig 28:**
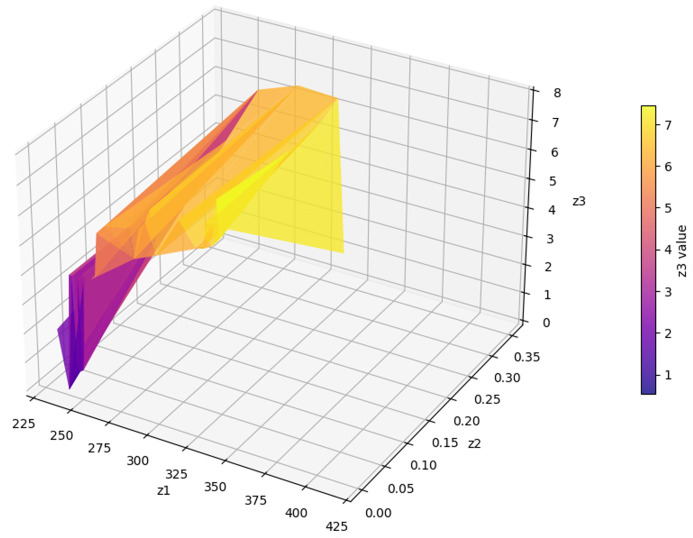
Three-dimensional stem plot of the space solution (pareto front).

**Fig 29:**
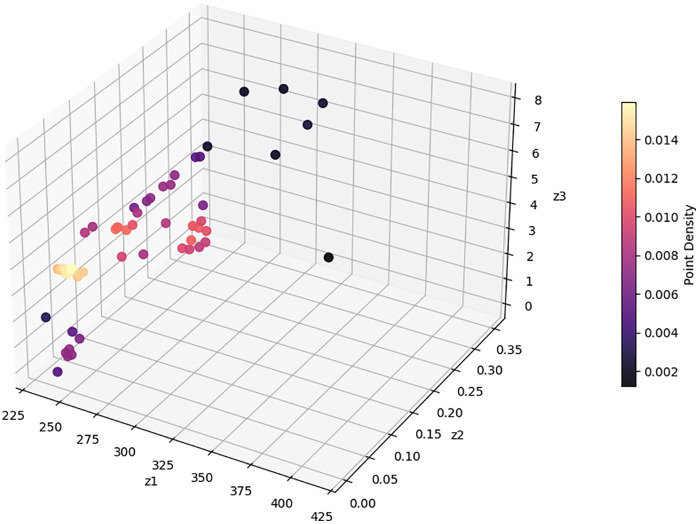
Point density distribution of pareto front in the space solution.

Accordingly, Figs 28 and 29 present the three-dimensional Pareto front in the solution space and the corresponding point density, illustrating the model’s behavior in terms of achieving lower cost ($Z_{\text{1}},Z_{\text{2}}$) while maximizing customer satisfaction ($Z_{\text{3}})$.

For more details, Table 17 compares the correlation between each objective function and the emergency radius. Based on the results, Z1 and Z3 show the strongest correlation. Z1 and Z2 exhibit a moderate negative correlation, while Z2 and Z3 show the weakest correlation overall.

**Table 17:** The Pearson correlation between objective functions and the emergency radius.

Pearson correlations	Z1	Z2	Z3	re
Z1	1	−0.1399	0.7724	−0.2540
Z2	−0.1399	1	0.1098	0.3698
Z3	0.7724	0.1098	1	−0.1163
Re	−0.2540	0.3698	−0.1163	1

### The relation between objective functions and the emergency radius

**Fig 30:**
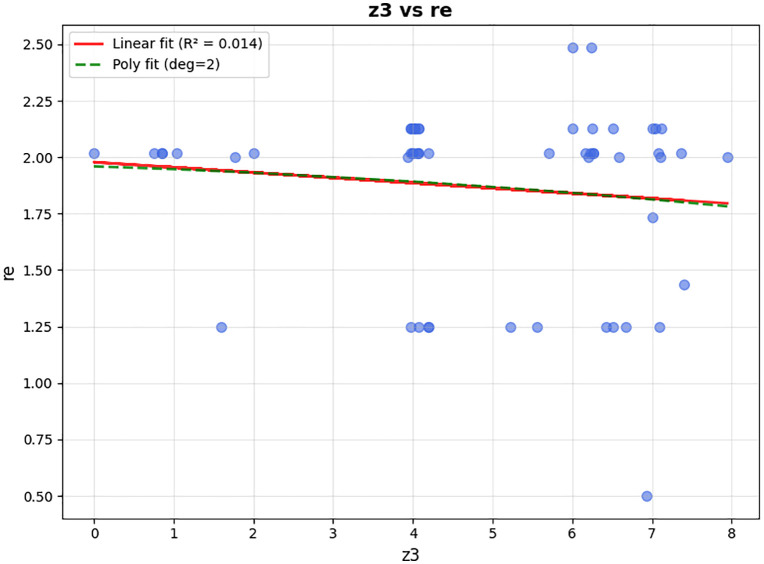
The relation between emergency radius (re) and patients’ satisfaction level (Z3).

**Fig 31:**
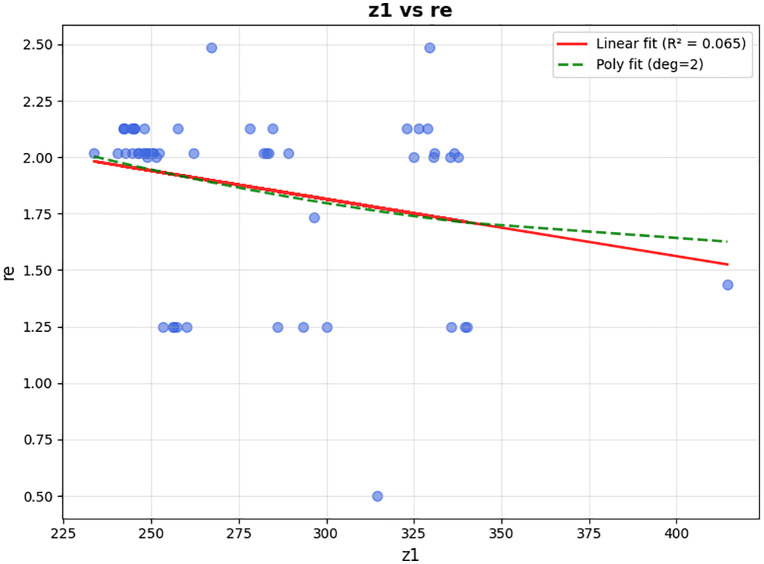
The relation between the emergency radius (re) and the first objective function (Z1).

**Fig 32:**
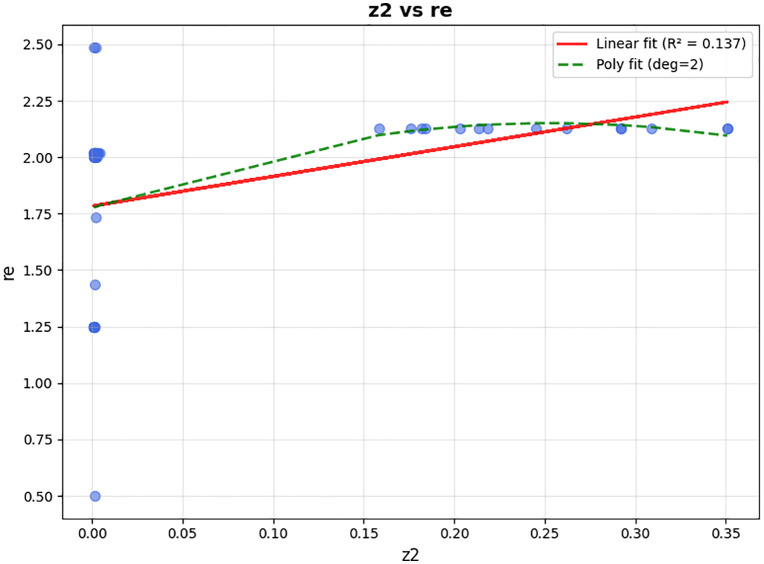
The relation between the emergency radius (re) and the first objective function (Z2).

As shown in Fig 30, the level of patient satisfaction ($Z_{\text{3}}$) has decreased with the increase in emergency coverage radius (*re*). In Fig 31, the economic costs ($Z_{\text{1}}$) are decreasing clearly with the increase in the emergency radius. Because as the radius increases, more patients are assigned to the AML, and the number of traveling nodes reduces. In Fig 32, we observe a direct relationship between $Z_{\text{2}}$ and *re*. At first glance, this may seem counterintuitive: increasing *re* allocates more patients to AML, which should reduce the number of trips. However, this highlights an important effect beyond the trip count. As *re* increases, customer satisfaction (CS) tends to decrease. To compensate this drop, the model prioritizes on-time delivery within the time windows, even if that requires faster and sometimes longer-distance routes instead of shortest paths. As a result, complying with time windows can lead to routes that incur longer travel distances. Overall, the model reflects a trade-off where CS has greater weight than GHG emissions. Therefore, it favors speed and schedule compliance, even if that entails higher travel costs (which to offset that, the model may choose inferior fuel types) and greater emissions to better meet CS objectives. We can observe these relationships numerically in Table 17. Based on the results, $Z_{\text{2}}$ and re show the strongest positive correlation. In addition, *re* has a moderate negative correlation with the other objective functions.

## Conclusion and future research

The delivery of medications has become increasingly important, but there are still few studies that have solved the logistics problem of medication delivery while considering potential facility locations and vehicle routing. In this study, a sustainable medication distribution system was proposed through AMLs and home delivery, which prioritized patient satisfaction and considered GHG emissions, covering radius, and departure time as decision variables. This model is the first comprehensive approach that covers all sustainability aspects for medication delivery. Our experimental results show that using AMLs reduces costs by 14.742% and 13.511% in different scenarios. However, patient satisfaction is negatively impacted, and there is a trade-off between the first and third objectives. The proposed model significantly improves the performance of home healthcare, especially in lockdown conditions. While GAMS provides an accurate solution, the NSGA-II algorithm is preferred as it requires less time and provides a good solution. Therefore, the use of the NSGA-II algorithm is recommended in most cases, except when an optimal and accurate solution is required and the solution time is not a critical factor.

In future research, it is suggested to focus on travel time uncertainty and road congestion, as well as to model the problem as a multi-depot system, taking into account transshipment and periodic service ordering. These issues are essential to improving the performance of medication delivery systems and making them more sustainable. Moreover, the path is still open to further study of triage with more than two levels and integration by a data-driven recommender system. Additionally, integrating RL-based techniques such as the Q-learning approach of Liu et al. [54] into multi-objective LRPs, can be a promising research avenue for the future.

## Supporting information

S1 File Additional details for the formulation of the second scenario and linearization approach. (PDF)
