# Supplementary material for: Optimizing sustainable healthcare location routing problem: Incorporating triage, automated medicine lockers, and soft time windows
Source: PLoS One. 2026 May 29;21(5):e0349445. doi: 10.1371/journal.pone.0349445 (PMC13221154; doi:10.1371/journal.pone.0349445)
Supplement: S1 File — (PDF) [file pone.0349445.s001.pdf]

Supplementary material

A. Mathematical formulation for the second scenario

$$Min\ Z1 = \sum_{k' \in K'} \sum_{i \in O \cup P^n \cup P^e \cup L} \sum_{j \in O+1 \cup P^n \cup P^e \cup L} fc^{tf}.\delta^{tf,k'}.d_{ij}.F_{ij}^{k'r}.x_{ij}^{k'} + \sum_{k' \in K'} G(c_w, c_a, \lambda_{n+1}^{k'}, \lambda_0^{k'}) + \sum_{k' \in K'} \sum_{i \in O \cup P^n \cup P^e \cup L} \sum_{j \in O+1 \cup P^n \cup P^e \cup L} tl_{ij} x_{ij}^{k'} \quad \text{(A-1)}$$

$$Min\ Z2 = \sum_{i \in O \cup P^n \cup P^e \cup L} \sum_{j \in O+1 \cup P^n \cup P^e \cup L} \sum_{k' \in K'} \sum_{tf \in tf} \delta^{tf,k'}.ef^{co2,tf}.d_{ij}.F_{ij}^{k'r}.x_{ij}^{k'} \quad \text{(A-2)}$$

$$Max\ Z3 = \sum_{i \in P^n \cup P^e} \sum_{k' \in K'} \mathrm{Cs}^p(t_{ik'}) + \sum_{j \in P^n \cup P^e\ allocated\ to\ lockers} \sum_{k' \in K'} \mathrm{Cs}^l(t_{jk'}) \quad \text{(A-3)}$$

St:

$$\sum_{j \in L} u_{ij} = z_i \qquad \forall i \in P^n \cup P^e \quad \text{(A-4)}$$

$$w_i + z_i = 1 \qquad \forall i \in P^n \cup P^e \quad \text{(A-5)}$$

$$u_{ij} \le v_j \qquad \forall i \in P^n \cup P^e, j \in L \quad \text{(A-6)}$$

$$u_{ij} d_{ij} \le r_i^n \qquad \forall i \in P^n, j \in L \quad \text{(A-7)}$$

$$u_{ij} d_{ij} \le r_i^e \qquad \forall i \in P^e, j \in L \quad \text{(A-8)}$$

$$r_j^e v_j \le d_{ij} + z_i M \qquad \forall i \in P^e, j \in L \quad \text{(A-9)}$$

$$r_j^n v_j \le d_{ij} + z_i M \qquad \forall i \in P^n, j \in L \quad \text{(A-10)}$$

$$\sum_{i \in P^n \cup P^e \cup O \cup L} x_{ijk'} = \sum_{i \in P^n \cup P^e \cup O \cup L} x_{jik'} = y_i^{k'} \qquad \forall j \in P^n \cup P^e \cup L, k' \in K' \quad \text{(A-11)}$$

$$x_{Ojk'} = x_{jO+1k'} = y_O^{k'} \qquad \forall j \in P^n \cup P^e \cup L, k' \in K' \quad \text{(A-12)}$$

$$\sum_{k'} y_i^{k'} = w_i \qquad \forall i \in P^n \cup P^e \cup L \quad \text{(A-13)}$$

$$x_{ijk'} \left(t_{ik'} + s_i + \sum_{r \in R} (\frac{d_{ij}}{v^r}) w_{ij}^{k'r} - t_{jk'} \right) \le 0 \qquad \forall i \in P^n \cup P^e \cup L \cup \{O\}, j \in P^n \cup P^e \cup L, k' \in K \quad \text{(A-14)}$$

$$G\left(c_w, c_a, \lambda_{n+1}^{k'}, \lambda_o^{k'}\right) = \begin{cases} \sum_{k'} c_w(\lambda_{n+1}^{k'} - 8) + c_a(8 - \lambda_o^{k'}), & \lambda_o^{k'} \le 8 \le \lambda_{n+1}^{k'} \le 18 \\ \sum_{k'} c_a(\lambda_{n+1}^{k'} - \lambda_o^{k'} - 10) + 10c_w, & \lambda_o^{k'} \le 8 \le 18 \le \lambda_{n+1}^{k'} \\ \sum_{k'} c_w(\lambda_{n+1}^{k'} - \lambda_o^{k'}), & 8 \le \lambda_o^{k'} \le \lambda_{n+1}^{k'} \le 18 \\ \sum_{k'} c_w(18 - \lambda_{n+1}^{k'}) + c_a(\lambda_o^{k'} - 18), & 8 \le \lambda_o^{k'} \le 18 \le \lambda_{n+1}^{k'}\} \end{cases} \quad \text{(A-15)}$$

$$\lambda_o^{k'} \ge a' \qquad \forall k' \in K' \quad \text{(A-16)}$$

$$\lambda_{n+1}^{k'} \le b' \qquad \forall k' \in K' \quad \text{(A-17)}$$

$$\sum_r w_{ij}^{rk'} = 1 \qquad \forall k' \in K', (i,j) \in A \quad \text{(A-18)}$$

$$Lv_{ij}^{k'} \le \sum_r v^r\, w_{ij}^{rk'} \le Uv_{ij}^{k'} \qquad \forall k' \in K', (i,j) \in A \quad \text{(A-19)}$$

$$r_j^n \ge r_j^e \qquad \forall j \in L \quad \text{(A-20)}$$

$$\begin{array}{l} w_{ij}^{rk\prime}, y_{ik}, x_{ijk}, v_i, u_{ij}, z_i, w_i \in \{0,1\} \\ \lambda_o^{k'}, \lambda_{n+1}^{k'}, t_{ik} \ge 0 \end{array} \quad \text{(A-21)}$$

B. Linearization

1) Linearization of the objective functions

To linearize the multiplication of two binary and continuous variables in the first part of the first objective function, which is to minimize the cost of fuel consumption, and in the second objective function, to minimize the GHGs emissions according to the amount of fuel consumption, which variable $x_{ij}^{k\prime}$ is multiplied by the variable $w_{ij}^{rk'}$ formulated in the fuel consumption function $F_{ij}^{k'r}$, the following restrictions are added:

$$x_{ij}^{k'}.F_{ij}^{k'r} = x_{ij}^{k'}.w'^{k',r}_{i,j}\lambda(kf.N.v + \omega\lambda\alpha.v_r + \beta\gamma.v_r^3).\frac{\bar{d}_{ij}}{v_r} \quad \text{(B-1)}$$

$$\omega_{ij}^{k\prime r} = x_{ij}^{k'}.F_{ij}^{k'r} \qquad \forall i \in P^n \cup P^e \cup \{O\}, j \in P^n \cup P^e \cup \{0+1\}, k' \in K', r \in R \quad \text{(B-2)}$$

$$\omega_{ij}^{k\prime r} \le F_{ij}^{k'r} \qquad \forall i \in P^n \cup P^e \cup \{O\}, j \in P^n \cup P^e \cup \{0+1\}, k' \in K', r \in R \quad \text{(B-3)}$$

$$\omega_{ij}^{k\prime r} \le M.x_{ij}^{k'} \qquad \forall i \in P^n \cup P^e \cup \{O\}, j \in P^n \cup P^e \cup \{0+1\}, k' \in K', r \in R \quad \text{(B-4)}$$

$$\omega_{ij}^{k\prime r} \geq F_{ij}^{k'r} - M\left(1 - x_{ij}^{k'}\right) \qquad \forall i \in P^n \cup P^e \cup \{O\}, j \in P^n \cup P^e \cup \{0+1\}, k' \in K', r \in R \qquad \text{(B-5)}$$

$$\omega_{ij}^{k\prime r} \geq 0 \qquad \text{(B-6)}$$

2) Linearization of time window constraints

Constraints (A-14) and (A-15) are related to the traveling time of vehicles on the routes leading to patients or lockers, with the implementation of these restrictions, the arrival time of vehicles to each destination is calculated. Considering that the binary variable x_{ij}^{k} is multiplied by the continuous variable of the vehicle's arrival time at each destination and the binary variable speed level selection, it is necessary to linearize these two constraints. So, we will have:

$$x_{ijk}\left(t_{ik} + s_i + \sum_{r\in R}(\frac{\bar{d}_{ij}}{v^r})w_{ij}^{kr} - t_{jk}\right) \leq 0 \qquad \forall i \in P^n \cup P^e \cup \{O\}, j \in P^n \cup P^e, k \in K \qquad \text{(B-7)}$$

$$x_{ijk}.t_{ik} + x_{ijk}.s_i + \sum_{r\in R}(\frac{\bar{d}_{ij}}{v^r})w_{ij}^{kr}.x_{ijk} - t_{jk}.x_{ijk} \leq 0 \qquad \text{(B-8)}$$

$$\mu_{ijk} = x_{ijk}.t_{ik} \qquad \text{(B-9)}$$

$$\mu_{ijk} \leq t_{ik} \qquad \forall i \in P^n \cup P^e \cup \{O\}, j \in P^n \cup P^e, k \in K \qquad \text{(B-10)}$$

$$\mu_{ijk} \leq M.x_{ijk} \qquad \forall i \in P^n \cup P^e \cup \{O\}, j \in P^n \cup P^e, k \in K \qquad \text{(B-11)}$$

$$\mu_{ijk} \geq t_{ik} - M.(1 - x_{ijk}) \qquad \forall i \in P^n \cup P^e \cup \{O\}, j \in P^n \cup P^e, k \in K \qquad \text{(B-12)}$$

$$\mu_{ijk} \geq 0 \qquad \text{(B-13)}$$

$$\rho_{ij}^{kr} = w_{ij}^{kr}.x_{ijk} \qquad \text{(B-14)}$$

$$\rho_{ij}^{kr} \leq x_{ijk} \qquad \text{(B-15)}$$

$$\rho_{ij}^{kr} \leq w_{ij}^{kr} \qquad \text{(B-16)}$$

$$\rho_{ij}^{kr} \geq w_{ij}^{kr} + x_{ijk} - 1 \qquad \text{(B-17)}$$

$$\rho_{ij}^{kr} \in \{0,1\} \qquad \text{(B-18)}$$

Therefore, constraint (A-14) is written as follows:

$$\mu_{ijk} + x_{ijk}.s_i + \sum_{r\in R}(\frac{\bar{d}_{ij}}{v^r})\rho_{ij}^{kr} - \mu_{jk} \leq 0 \qquad \text{(B-19)}$$

$$\mu_{ijk} \leq t_{ik} \qquad \forall i \in P^n \cup P^e \cup \{O\}, j \in P^n \cup P^e, k \in K \qquad \text{(B-20)}$$

$$\mu_{ijk} \leq M.x_{ijk} \qquad \forall i \in P^n \cup P^e \cup \{O\}, j \in P^n \cup P^e, k \in K \tag{B-21}$$

$$\mu_{ijk} \geq t_{ik} - M.(1 - x_{ijk}) \qquad \forall i \in P^n \cup P^e \cup \{O\}, j \in P^n \cup P^e, k \in K \tag{B-22}$$

$$\rho_{ij}^{kr} \leq x_{ijk} \tag{B-23}$$

$$\rho_{ij}^{kr} \leq w_{ij}^{kr} \tag{B-24}$$

$$\rho_{ij}^{kr} \geq w_{ij}^{kr} + x_{ijk} - 1 \tag{B-25}$$

$$\rho_{ij}^{kr} \in \{0,1\}, \mu_{ijk} \geq 0 \tag{B-26}$$

We will have the same steps for constraint (A-15).

3) Linearization of driver's wage cost

The seventh part of the first objective function, which the G function represents, calculates the cost of the driver's wages. If the drivers start working earlier than the scheduled time, which is 8:00 a.m., and also, they finish working later than the determined time, which is 6:00 p.m., they will be subject to overtime and higher wages than the normal hourly rate. Constraint (A-16) expresses these different situations of wages. This nonlinear constraint is linearized as follows:

$$G\left(c_w, c_a, \lambda_{n+1}^{k'}, \lambda_o^{k'}\right) \geq \sum_{k'} c_w(\lambda_{n+1}^{k'} - 8) + c_a(8 - \lambda_o^{k'}) - M(1 - \upsilon_1) \tag{B-27}$$

$$G\left(c_w, c_a, \lambda_{n+1}^{k'}, \lambda_o^{k'}\right) \leq \sum_{k'} c_w(\lambda_{n+1}^{k'} - 8) + c_a(8 - \lambda_o^{k'}) + M(1 - \upsilon_1) \tag{B-28}$$

$$G\left(c_w, c_a, \lambda_{n+1}^{k'}, \lambda_o^{k'}\right) \geq \sum_{k'} c_w(\lambda_{n+1}^{k'} - \lambda_o^{k'}) - M(1 - \upsilon_2) \tag{B-29}$$

$$G\left(c_w, c_a, \lambda_{n+1}^{k'}, \lambda_o^{k'}\right) \leq \sum_{k'} c_w(\lambda_{n+1}^{k'} - \lambda_o^{k'}) + M(1 - \upsilon_2) \tag{B-30}$$

$$\left(c_w, c_a, \lambda_{n+1}^{k'}, \lambda_o^{k'}\right) \geq \sum_{k'} c_w(18 - \lambda_o^{k'}) + c_a(\lambda_{n+1}^{k'} - 18) - M(1 - \upsilon_3) \tag{B-31}$$

$$\left(c_w, c_a, \lambda_{n+1}^{k'}, \lambda_o^{k'}\right) \leq \sum_{k'} c_w(18 - \lambda_o^{k'}) + c_a(\lambda_{n+1}^{k'} - 18) + M(1 - \upsilon_3) \tag{B-32}$$

$$G\left(c_w, c_a, \lambda_{n+1}^{k'}, \lambda_o^{k'}\right) \geq \sum_{k'} c_a(\lambda_{n+1}^{k'} - \lambda_o^{k'} - 10) + 10c_w - M(1 - \upsilon_4) \tag{B-33}$$

$$\left(c_w, c_a, \lambda_{n+1}^{k'}, \lambda_o^{k'}\right) \leq \sum_{k'} c_a(\lambda_{n+1}^{k'} - \lambda_o^{k'} - 10) + 10c_w + M(1 - \upsilon_4) \tag{B-34}$$

$$\lambda_o^{k'} \leq 8 + M(1 - \upsilon_1) \tag{B-35}$$

$$\lambda_{n+1}^{k'} \geq 8 - M(1 - \upsilon_1) \tag{B-36}$$

$$\lambda_{n+1}^{k'} \leq 18 + M(1 - \upsilon_1) \tag{B-37}$$

$$\lambda_o^{k'} \geq 8 - M(1 - \upsilon_2) \tag{B-38}$$

$$\lambda_o^{k'} \leq \lambda_{n+1}^{k'} + M(1 - \upsilon_2) \tag{B-39}$$

$$\lambda_{n+1}^{k'} \leq 18 + M(1 - \upsilon_2) \tag{B-40}$$

$$\lambda_o^{k'} \geq 8 - M(1 - \upsilon_3) \tag{B-41}$$

$$\lambda_o^{k'} \leq 18 + M(1 - \upsilon_3) \tag{B-42}$$

$$\lambda_{n+1}^{k'} \geq 18 - M(1 - \upsilon_3) \tag{B-43}$$

$$\lambda_o^{k'} \leq 8 + M(1 - \upsilon_4) \tag{B-44}$$

$$\lambda_{n+1}^{k'} \geq 18 - M(1 - \upsilon_4) \tag{B-45}$$

$$\upsilon_1 + \upsilon_2 + \upsilon_3 + \upsilon_4 = 1 \tag{B-46}$$

$$\upsilon_i \in \{0,1\} \tag{B-47}$$

The G function calculates the driver's wage cost in the first objective function. Therefore, the function G is equal to one of the criteria of the function based on the working hours. We have shown this equality as two smaller and bigger constraints. This concept is expressed by the pairs of constraints (B-27) and (B-28), (B-29) and (B-30), (B-31) and (B-32), (B-33) and (B-34). The rest constraints state the conditions of each criterion. For example, constraints (B-35), (B-36), and (B-37) are written for the first criterion of function G. Constraint (B-35) states that if the departure time of the driver from the depot is less than 8 (overtime), and constraints (B-36) and (B-37) state that if the time of finishing work is less than 18, i.e., at the normal hour, the first criterion must be followed, which is done by adding a binary variable (constraint (B-47)). Only one binary variable should be selected among others (constraint (B-46)). Similarly, constraints (B-38), (B-39), and (B-40) are for the activation of the second criterion, constraints (B-41), (B-42), and (B-43) are for the activation of the third criterion, and constraints (B-44), (B-45), and (B-46) are for the fourth criterion.

4) Linearization of the emergency radius limit

This constraint, which is stated in line 6 of the mathematical model, is the product of a variable binary υ_1 in the continuous variable related to the emergency radius. Its linearization is as follows:

$\tau_j \leq d_{ij} + z_i . M$ $\quad \forall j \in L, i \in P^e$ (B-48)

$\tau_j \leq r_j^e$ $\quad \forall j \in L$ (B-49)

$\tau_j \leq v_j . M$ $\quad \forall j \in L$ (B-50)

$\tau_j \geq r_j^e - (1 - v_j) . M$ $\quad \forall j \in L$ (B-51)

Constraint (B-48) expresses the same constraint of line 6 of the mathematical model, where the new variable τ is considered instead of the prior product. Constraint (B-49) states that the value of this variable must be less than the emergency radius. Constraint (B-50) states that it is possible to quantify this variable only if the patient is allocated to a locker, and also constraint (B-51) expresses that τ must be equal to the emergency coverage radius if the patient is allocated to the locker.

5) Linearization of patients' satisfaction function in the third objective function

As explained earlier, this function consists of 4 linear functions, the value of which is determined according to the vehicle arrival time at the patient (t_{ik}), which is one of the decision variables of the problem. The third objective function includes the sum of all the values that this function determines for the delivery time to all patients. Therefore, it is necessary to linearize it. Therefore, for each $\forall i \in P^n \cup P^e \cup \{O\}, k \in K$ we have:

$Cs\ (t_{ik}) \geq -M(1 - \eta_1)$ (B-52)

$Cs\ (t_{ik}) \leq M(1 - \eta_1)$ (B-53)

$Cs\ (t_{ik}) \geq \frac{t_{ik} - EET}{PET - EET} - M(1 - \eta_2)$ (B-54)

$Cs\ (t_{ik}) \leq \frac{t_{ik} - EET}{PET - EET} + M(1 - \eta_2)$ (B-55)

$Cs\ (t_{ik}) \leq 1 + M(1 - \eta_3)$ (B-56)

$s\ (t_{ik}) \geq 1 - M(1 - \eta_3)$ (B-57)

$Cs\ (t_{ik}) \leq \frac{ELT - t_{ik}}{ELT - PLT} + M(1 - \eta_4)$ (B-58)

$Cs\ (t_{ik}) \geq \frac{ELT - t_{ik}}{ELT - PLT} - M(1 - \eta_4)$ (B-59)

$t_{ik} \leq EET + M(1 - \eta_1)$ (B-60)

$t_{ik} \geq ELT - M(1 - \eta_1)$ (B-61)

$t_{ik} \geq EET - M(1 - \eta_2)$ (B-62)

$$t_{ik} \leq PET + M(1 - \eta_2) \quad \text{(B-63)}$$

$$t_{ik} \geq PET - M(1 - \eta_3) \quad \text{(B-64)}$$

$$t_{ik} \leq PLT + M(1 - \eta_3) \quad \text{(B-65)}$$

$$t_{ik} \geq PLT - M(1 - \eta_4) \quad \text{(B-66)}$$

$$t_{ik} \leq ELT + M(1 - \eta_4) \quad \text{(B-67)}$$

$$t_{ik} \leq ELT + M(1 - \eta_4) \quad \text{(B-68)}$$

$$\eta_i \in \{0,1\} \quad \text{(B-69)}$$

In the second scenario, considering that the problem may be used for situations where the cost of renting a vehicle is high, buying a vehicle is not possible for a long time, or due to the time limit, the vehicle has to travel the route of patients in addition to visiting the locker, we have suggested the integrated use of vehicles and we are examining the transportation cost. In some circumstances, even the answer to this question of which locker should be selected for replenishment may be affected, and the amount of fuel consumed may change depending on the use of one type of vehicle. In the previous case, it was required to use the second type of vehicle even in the case of using only one locker, while this is a limiting assumption that seems to be manageable by paying less money. However, the costs have been compared for a real example and explained in the following section. In this scenario, the items related to indices, parameters, and variables will remain in the same order, with the difference that the variables that distinguished the transport route and the vehicle for transferring the medicine to patients and lockers have been used in an integrated manner.
